# Colloidal Photonic Fibers for Reflectively Colorful Radiative Cooling Fabrics

**DOI:** 10.1002/smsc.70273

**Published:** 2026-04-21

**Authors:** Sewon Ahn, Jaewon Lee, Soyul Kwak, Eunji Im, YongDeok Cho, Hyeon Ho Kim, Heon Lee, Seungwoo Lee

**Affiliations:** ^1^ KU‐KIST Graduate School of Converging Science and Technology Korea University Seoul Republic of Korea; ^2^ Department of Biomicrosystem Technology Korea University Seoul Republic of Korea; ^3^ Department of Materials Science and Engineering Korea University Seoul Republic of Korea; ^4^ Department of Integrative Energy Engineering Korea University Seoul Republic of Korea; ^5^ Center for Opto‐Electronic Materials and Devices Post‐Silicon Semiconductor Institute Korea Institute of Science and Technology (KIST) Seoul Republic of Korea

**Keywords:** colloids, metamerism, photonic glass, radiative cooling, structured fluids

## Abstract

Daytime radiative cooling has emerged as a promising passive strategy for sustainable thermal management, yet its widespread implementation remains hindered by the lack of colorful, transformable, and scalable material systems. Here, we present a new class of structurally colored radiative cooling fibers created by microfluidic extrusion of mid‐infrared (mid‐IR)–emissive colloidal structural fluids. These fluids consist of monodisperse silica (SiO_2_) nanoparticles (NPs) suspended in an acrylate resin, which self‐assemble into amorphous photonic glass during microfluidic shear flow and are subsequently solidified by in situ photocuring. The resulting fibers exhibit angle‐independent and permanent structural coloration across the visible spectrum, which is tunable via NP size and interparticle spacing without the use of pigments or dyes while maintaining broadband mid‐IR emissivity (>0.9) arising from SiO_2_ phononic vibrations. When woven into fabrics, these photonic fibers combine high solar reflectance with strong mid‐IR thermal emission, enabling effective reduction of solar heat gain and daytime cooling of a heated skin‐mimicking substrate even under direct sunlight. Outdoor tests confirm that photonic glass textiles achieve a large temperature depression on simulated skin phantoms, consistent with their broad visible scattering and high mid‐IR emissivity. This work introduces a scalable and esthetically versatile route toward colorful radiative‐cooling textiles, bridging photonic design with practical energy‐saving applications in outdoor and wearable systems.

## Graphical abstract

Microfluidic extrusion of mid‐infrared (IR)–emissive silica colloidal structural fluids enables scalable photonic glass fibers with angle‐independent, pigment‐free structural colors tunable across the visible spectrum while maintaining broadband mid‐IR emissivity (>0.9). Woven into fabrics, these fibers combine high solar reflectance and strong thermal emission to deliver pronounced daytime radiative cooling under direct sunlight in outdoor tests.

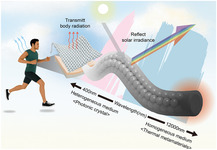

## 1 Introduction

As global warming accelerates, radiative cooling technologies, particularly including daytime radiative cooling, are becoming increasingly important. The key to daytime radiative cooling lies in maximizing emissivity and absorptivity in the mid‐infrared (mid‐IR) regime, while simultaneously enhancing broadband solar reflectivity. Consequently, since the pioneering report of daytime radiative cooling [1, 2, 3], early research efforts have primarily focused on developing whitish radiative cooling materials. However, colorization of radiative cooling materials has recently emerged as a critical requirement for enabling transformative and more practical applications [4, 5, 6, 7, 8, 9, 10, 11, 12, 13, 14, 15, 16, 17, 18, 19]. In particular, most of the reported colored radiative cooling systems have relied on molecular dyes or pigments to achieve visible coloration [9, 11, 18, 20, 21], yet such absorption‐based coloration inevitably suffers from photobleaching or fading under prolonged solar irradiation. This limitation poses a fundamental obstacle for long‐term outdoor operation, where intense UV exposure continuously degrades dye molecules and compromises both color appearance and cooling efficiency.

In parallel, diversification of material platforms beyond the conventional flat ceramic or polymeric film has become essential [22, 23, 24, 25, 26], particularly for outdoor use. Reflecting this trend, radiative cooling materials are evolving toward more versatile forms, including particulate coating paints [13, 26, 27, 28, 29], fibers, and woven fabrics [8, 30, 31, 32, 33, 34]. Furthermore, a few pioneering studies have recently begun to integrate structural coloration into radiative cooling fabrics to ensure long term esthetic and thermal stability [35, 36, 37, 38]. For instance, noniridescent structural colorants based on core–shell microspheres have been applied to fabric surfaces to achieve daytime cooling and Janus‐structured amorphous photonic crystal textiles have been developed for dual‐mode thermal regulation [37, 38]. While these studies demonstrate the viability of structurally colored radiative cooling at the textile scale, most current approaches rely on coating structural colorants onto existing fabrics by dip‐coating or spray‐casting. Such extrinsic modifications may encounter certain practical hurdles; for instance, the colored layers can be susceptible to mechanical damage, such as delamination, cracking, or fading during repeated wear and washing, which could eventually compromise both the optical appearance and cooling performance.

In this work, we propose that mid‐IR‐emissive colloidal structural fluids capable of exhibiting structural colors can be engineered into fiber architectures using microfluidic extrusion to create colorful radiative cooling textiles. By harnessing the spontaneous self‐assembly of mid‐IR‐emissive silica (SiO_2_) colloidal nanoparticles (NPs) into amorphous photonic glass within a photocurable resin, the microfluidically extruded and cured fibers exhibit angle‐independent and tunable visible structural coloration, which is controlled by interparticle spacing. Unlike existing methods that apply structural colors as external coatings, our approach embeds the photonic functionality directly within the fiber architecture. This strategy allows for a seamless transition from fluid to fiber, and finally to woven textiles, ensuring permanent color stability. Simultaneously, these fibers achieve high and broadband mid‐IR emissivity, enabled by the strong phononic vibrations of SiO_2_ NPs in the mid‐IR range [39]. These structurally colored fibers not only retain the essential thermal management functionality required for daytime radiative cooling, including the ability to passively cool solar absorptive surfaces even when they are warmer than the ambient environment (artificial skin model), but also overcome the esthetic, material, and durability limitations of previously reported fiber systems. To provide a rigorous framework for scaling these individual fibers into macroscopic textiles, we also introduce a fiber‐to‐textile scale optical modeling workflow that quantitatively bridges the gap between individual fiber scattering and collective textile performance. Collectively, this strategy offers a scalable and versatile platform for the next incarnation of radiative cooling textiles, paving the way for their practical implementation in diverse outdoor and wearable applications.

## 2 Results and Discussion

### 2.1 Fabrication and Key Concept

**FIGURE 1:**
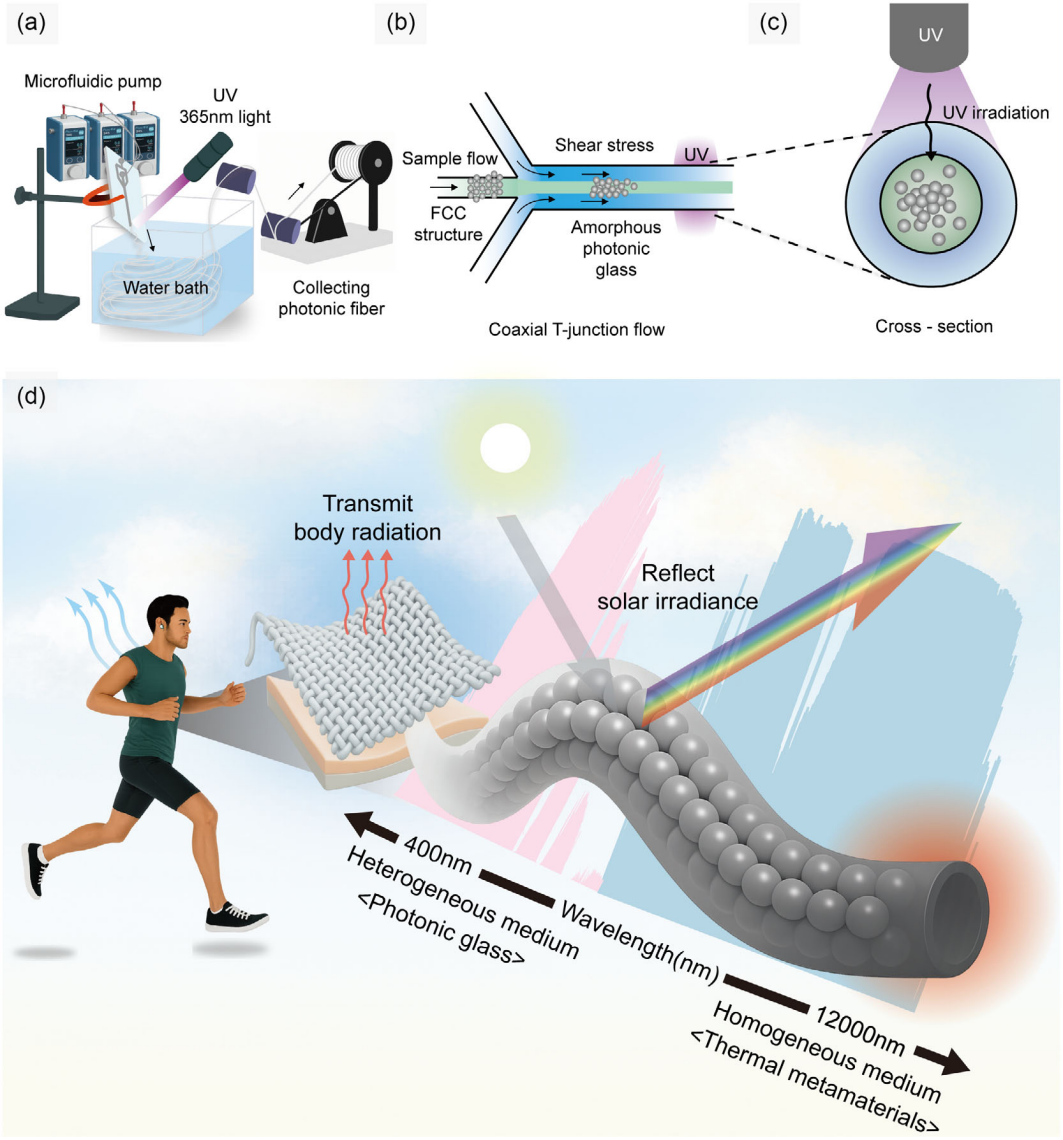
(a)–(c) Schematic of the microfluidic extrusion process for SiO_2_ colloidal fibers. Monodisperse SiO_2_ nanoparticles (NPs) are dispersed in an acrylate‐based resin (ETPTA) and extruded through a microfluidic T‐junction. Shear flow induces disordering of colloidal packing, and in situ UV curing solidifies the amorphous photonic glass fiber. (d) Design principle of dual‐band optical behavior. Disordered SiO_2_ assemblies produce angle‐independent visible structural coloration via multiple scattering, while the SiO_2_/ETPTA composite exhibits broadband mid‐IR emissivity (*ε* > 0.9) from phononic vibrations, enabling efficient solar reflection and thermal radiation for passive cooling.

Figure 1a–c outlines the key fabrication steps of our structurally colored radiative cooling fiber. SiO_2_ colloidal NPs can spontaneously assemble within an acrylate‐based liquid resin (Trimethylolpropane ethoxylate triacrylate, ETPTA), as their van der Waals interactions are effectively canceled due to the nearly identical dielectric constants between SiO_2_ and ETPTA (Figure S1) [40, 41]. This dielectric matching removes attractive interparticle forces, allowing uniformly sized SiO_2_ NPs to undergo entropy‐driven packing into face‐centered‐cubic (FCC) lattices.

When these colloidal structural fluids are linearly extruded into fibers with diameters of 100–150 μm via a microfluidic T‐junction, shear forces near the fiber surface disrupt the ordered FCC lattice, transforming the structure into an amorphous photonic glass (Figure 1a,b). Immediate UV irradiation at the outlet of the microfluidic channel enables in situ photocuring (Figure 1c), solidifying the fiber. By adjusting the ratio of curing agents, the photocrosslinking density can be tuned, imparting elastic properties to the resulting fiber.

As illustrated in Figure 1d, these fibers exhibit multiscale photonic engineering optimized for daytime radiative cooling. In the visible regime, the 120–180 nm SiO_2_ NPs are arranged with ∼50 nm interparticle spacing (20 vol%) into a disordered photonic glass structure, enabling multiple scattering and interference through short‐range structural correlations [42, 43, 44]. This results in noniridescent, angle‐independent structural coloration that reflects solar radiation with minimal absorption, thereby enhancing radiative cooling performance, particularly under direct sunlight. In the mid‐IR regime, the SiO_2_ NPs fall well within the deep‐subwavelength limit. Consequently, the SiO_2_ NP–ETPTA composite functions as an effective homogeneous medium, with strong phononic vibrations of the SiO_2_ NPs giving rise to high and broadband mid‐IR emissivity. This dual‐band optical behavior (i.e., selective solar reflectance in the visible and strong thermal emission in the mid‐IR) enables passive heat rejection from the body or surrounding surfaces.

Moreover, these fiber architectures further amplify daytime radiative cooling performance by enhancing broadband solar light scattering. The 100–150 μm diameter fiber surfaces further contribute to direct scattering, while the interstitial voids between fibers promote multiple and broadband scattering events. Together, these effects reduce solar heat gain and maximize net radiative heat loss, positioning these photonic fibers as a powerful building block for next‐generation cooling textiles.

### 2.2 Photonic Design

**FIGURE 2:**
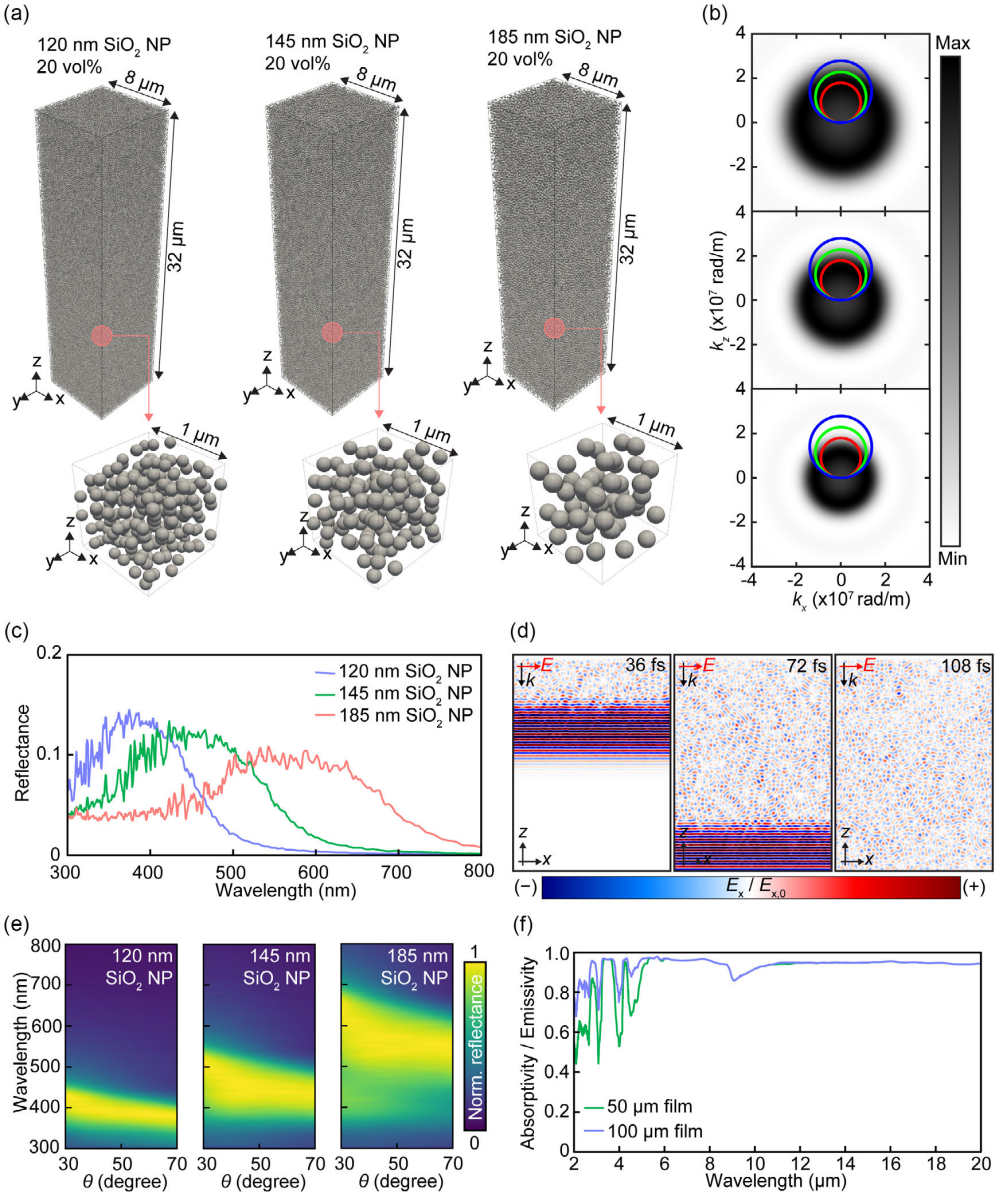
(a) Geometries of disordered SiO_2_ NP dispersions in an ETPTA medium used for finite‐difference time‐domain (FDTD) simulations. The insets highlight the random, amorphous packing of the NPs. (b) Calculated Ewald spheres in momentum (*k*)‐space representing the structural correlations of the photonic glass. (c) Numerically calculated (FDTD) reflectance spectra in the visible regime for the three SiO_2_ NP sizes. (d) Time‐traced electric field (*E*_*x*_) distribution for the 120 nm SiO_2_ NP‐ETPTA composite at a wavelength of 450 nm. (e) Angle‐resolved reflectance contour maps calculated with FDTD. The incident light angle was fixed at 13°, while the detection angle (*θ*) was varied. (f) Calculated broadband absorptivity/emissivity spectra of the SiO_2_‐ETPTA composite in the mid‐infrared (mid‐IR) range for film thicknesses of 50 and 100 µm.

To theoretically validate the multiscale photonic engineering outlined in Figure 1d, we performed ultrascale numerical simulations incorporating 452,800 SiO_2_ NPs (see more details in Figures S2 and S3). As mentioned earlier, the refractive index matching between SiO_2_ and ETPTA effectively minimizes interparticle van der Waals attractions. Consequently, the spatial arrangement is governed by short‐range repulsive forces, causing the colloids to behave as effective hard spheres with an effective diameter larger than their physical dimension. To model this system, we treated each NPs as a composite sphere consisting of a SiO_2_ core and an effective ETPTA shell. Using force‐biased and Lubachevsky–Stillinger algorithm, which reflect the spontaneous, entropically driven assembly of colloids into amorphous, densely packed configurations, these composite spheres were densely packed to a total volume fraction of ∼60% [45]. The thickness of ETPTA shell was precisely adjusted so that the SiO_2_ core fraction remained consistent with our experimental 20 vol%. These simulations allowed us to faithfully reconstruct the spatial arrangement and size distribution of colloidal photonic glasses within the fiber cross‐section. The calculated structural correlations and radial distribution functions confirmed the lack of long‐range order, consistent with the expected features of amorphous photonic media, as shown in theoretically expected Ewald sphere (Figure 2b). This realistic geometry was subsequently imported into full‐wave electromagnetic simulations using the FDTD method (MEEP 1.31) to evaluate both the visible‐range scattering behavior and the mid‐IR emissivity [46]. While T‐matrix‐based methods represent powerful and computationally efficient alternatives for multiple‐sphere scattering problems [47, 48], the FDTD approach provides a general full‐wave numerical framework applicable to our disordered slab geometry and enables direct access to time‐resolved field profiles used throughout this work.

The resulting optical spectra successfully predicted and validated key photonic design principles. As shown in Figure 2c, the composite exhibits broadband and noniridescent reflection in the visible regime, stemming from a combination of Mie scattering from colloidal motif (i.e., form factor) and resonant multiple scattering with short‐range structural correlations (i.e., structural factor). Starting with a flat SiO_2_ NP–ETPTA composite film, we observe that increasing the SiO_2_ NP size from 120 to 185 nm, while maintaining a fixed vol%, systematically redshifts the reflection peak, spanning across blue, green, and red wavelengths.

This color tunability arises from the interplay between two fundamental scattering mechanisms: the form factor, which reflects the individual scattering properties of each SiO_2_ NP, and the structure factor, which arises from short‐range correlations in the disordered photonic glass. As the NP size increases to target longer wavelengths, the form factor and structure factor become spectrally decoupled. As demonstrated in recent literature, this decoupling with an excessive multiple scattering background fundamentally limits the color purity and saturation for longer‐wavelength hues [49, 50]. Consequently, our reddish‐white composite exhibits a broadened reflection profile across most of the visible spectrum (from approximately 380–650 nm) rather than a sharp, narrow red peak. While this inherent spectral broadening limits hue selectivity and saturation, it is highly advantageous for daytime thermal management. This flattened, broadband reflection allows for a much more efficient rejection of solar irradiance, thereby improving the overall solar‐heat‐rejection performance of the textile and enhancing its daytime cooling effectiveness. Such contributions of form and structure factors to the reflected power spectrum can also be reproduced using a first‐order (Born type) approximation [42] based analytical analysis (Figure S4).

Figure 2d presents the electric field (E‐field) distribution at the resonant wavelength within a photonic glass composed of 120 nm SiO_2_ NPs, revealing light propagation in an intermediate regime between pure ballistic transport and diffusive multiple scattering. Due to the low refractive index contrast, the predominantly transmitted ballistic pulse is accompanied by relatively weak backward scattering (see also the far‐field angular distribution of the transmitted light in Figure S5). This correlated, weakly scattered light constructively interferes to produce structural coloration, while the low refractive index contrast suppresses the excessive multiple scattering that typically washes out structural color in high‐index contrast systems [50]. This behavior is further visualized in Supporting Movies S1‐S3. E‐field profiles for other NP sizes are summarized in Figure S6. Importantly, unlike photonic crystals such as FCC opals, which exhibit angle‐dependent iridescence, these amorphous photonic glasses yield angle‐independent colorization (Figure 2e), a key advantage for achieving uniform coloration across the curved surfaces of fibers.

As mentioned earlier, the SiO_2_ NP–ETPTA composite can be regarded as a homogeneous medium in the mid‐IR regime due to the subwavelength dimensions of the NP relative to thermal radiation wavelengths. Figure S7 shows the experimentally measured effective dielectric constants of a 20 vol% SiO_2_ NP–ETPTA composite. Based on these values, we numerically predict the mid‐IR absorptivity/emissivity of films with thicknesses of 50 and 100 μm. As shown in Figure 2f, even a 100 μm‐thick film can function as almost ideal broadband emitter, exhibiting absorptivity/emissivity values exceeding 0.9 across the mid‐IR spectrum. Notably, at a fixed vol%, the size of the SiO_2_ NP has negligible impact on mid‐IR absorptivity/emissivity, confirming that the composite behaves as an optically homogeneous thermal metamaterial in this spectral range.

**FIGURE 3:**
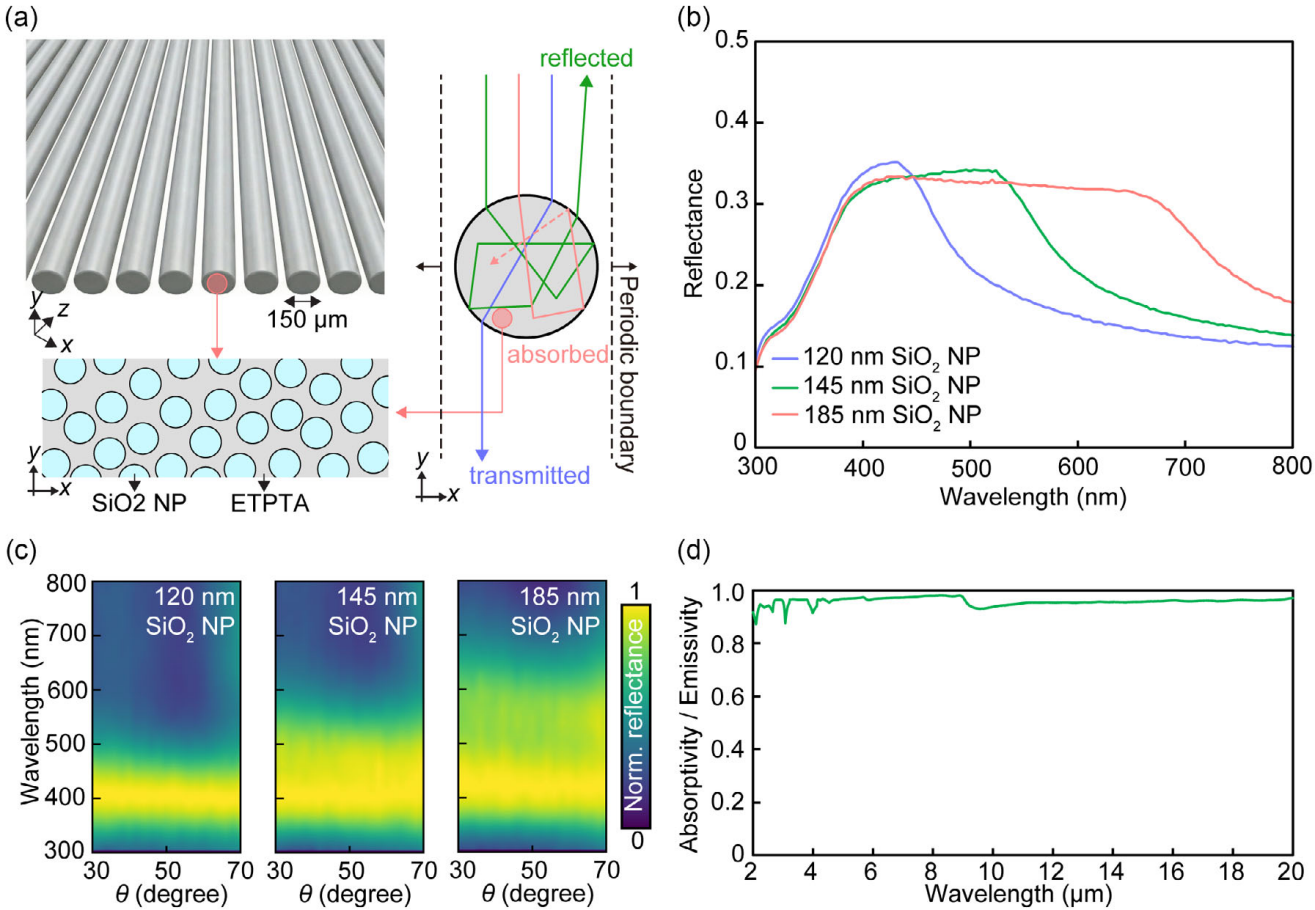
(a) Schematic illustration of the aligned fiber array (left) and the corresponding Monte Carlo simulation model (right) used for calculating optical properties of the photonic fiber. The model assumes that a fiber with a diameter of 150 µm is periodically arrayed. (b) Reflectance spectra of the fibers in the visible regime calculated using Monte Carlo simulations. The spectra confirm that the structural coloration is preserved within the fiber configuration. The coarse and fine roughness parameters are 0.9 and 0.1, respectively, throughout the Monte Carlo simulations for the fiber array. (c) Calculated angle‐resolved reflectance contour maps of the fibers as a function of *θ*, demonstrating the retention of angle‐independent coloration. The incident light angle was fixed at 13°. (d) Calculated broadband absorptivity/emissivity spectrum of the photonic glass fiber in the mid‐IR range.

While the FDTD simulations rigorously capture the light propagation through the photonic glass and the complex scattering phenomena of the constituent NPs, extending such full‐wave simulation to macroscopic fiber and textile architectures is computationally impractical due to the extreme memory and time requirements. To accurately evaluate the photonic performance of the material when engineered into fiber architectures, we employed Monte Carlo simulations on a periodically arrayed fiber model (diameter = 150 µm), as illustrated in Figure 3a. This simulation setup accounts for the complex light transport through curved surfaces and interstitial space while capturing the interplay between form and structure factors in the disordered photonic glass [44, 51]. This hybrid approach leverages the spectral characteristics derived from FDTD as a reliable benchmark to validate the Monte Carlo‐based large‐scale light transport model. Indeed, the Monte Carlo model yielded results highly consistent with the FDTD counterpart for the planar SiO_2_ NP–ETPTA composite (Figure S8). The resulting fiber spectra (Figure 3b) are highly consistent with the planar counterpart (Figure 2c), confirming that the structural coloration is fully preserved despite the geometric transition. The curvature and inter‐fiber voids promote multiple scattering events that enhance solar reflectance while maintaining the angle‐independence characteristic of photonic glass. As shown in Figure 3c, the isotropic scattering inherent to the amorphous structure dominates over geometric specular reflection, ensuring consistent coloration regardless of viewing angle. Furthermore, the fiber morphology does not compromise thermal management. The single‐layer array functions as a near‐ideal broadband thermal emitter with absorptivity/emissivity exceeding 0.9 across the mid‐IR range (Figure 3d), driven by multiple reflection and scattering events within and between the fibers. These results confirm that a single layer of photonic fibers is sufficient to realize dual‐band optical functionality, combining esthetic versatility with effective radiative cooling.

### 2.3 Microfluidic Synthesis of Photonic Fibers

**FIGURE 4:**
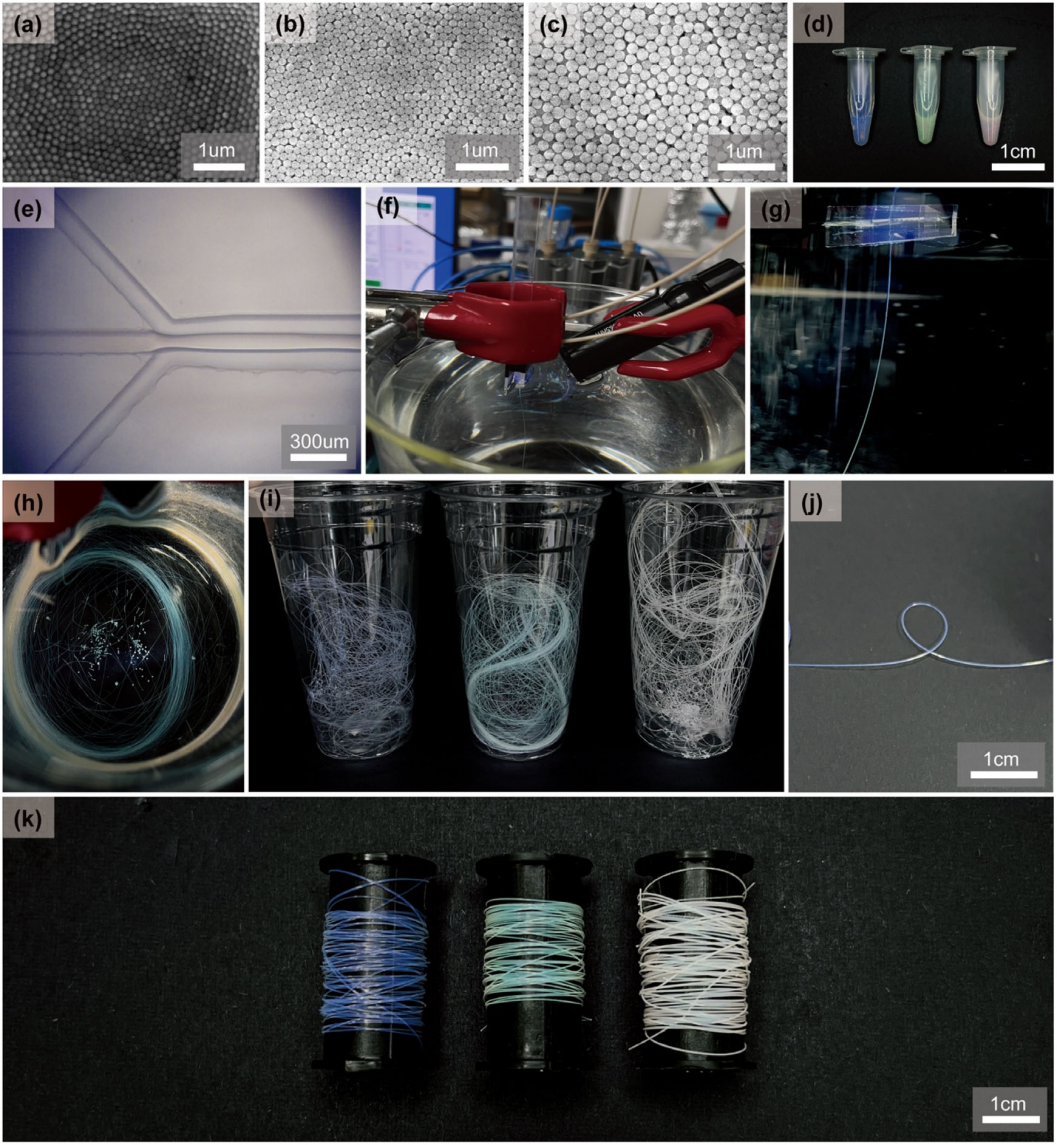
(a)–(c) SEM images of monodisperse SiO_2_ NPs synthesized by the Stöber method with controlled diameters of 120 (a), 150 (b), and 180 (c) nm, respectively. (d) Photographs of structural fluids prepared by dispersing each NP type in ETPTA liquid precursor, exhibiting structural colors of blue (120 nm), green (150 nm), and reddish‐white (180 nm). (e) OM image of a microfluidic T‐junction device, where aqueous sheath flows compress and thin the SiO_2_–ETPTA composite core stream. (f) Photograph of the microfluidic setup used for in situ ultraviolet (UV) photocuring of extruded composite fibers (optofluidic synthesis). (g,h) Continuous extrusion of solidified fibers collected in an aqueous bath and the subsequent entanglement of multicolored fibers after drying. (i) Bundled blue, green, and reddish‐white fibers exhibiting uniform structural coloration along their entire lengths. (j) Photograph showing the mechanical flexibility of a single cured fiber under loop formation without fracture. (k) Wound fiber spools displaying consistent color fidelity and scalability in packaging of radiative cooling threads.

We first synthesized highly uniform SiO_2_ NPs of different sizes using the Stöber method [52], as shown in Figure 4a–c. The synthesized SiO2 NPs were purified and dried for volume determination before being dispersed in the ETPTA liquid precursor at the target volume fraction as previously reported (Figure S9) [53, 54]. When these NPs were stably dispersed, the resulting structural fluids exhibited distinct reflective colors: blue for 120 nm NPs, green for 150 nm, and reddish‐white for 180 nm (Figure 4d).

Figure 4e shows an optical microscope (OM) image of the microfluidic T‐junction used in our study, where aqueous sheath flows apply shear forces and simultaneously thin the SiO_2_ NP–ETPTA core stream. Detailed chip design, fabrication, and operation OM images are provided in Figures S10–S12. At the outlet of the microfluidic chip, the extruded liquid fiber was exposed to ultraviolet (UV) light for in situ photocuring, as presented in Figure 4f (optofluidic synthesis). The resulting solidified fibers were continuously produced (Figure 4g) and collected in an aqueous bath (Figure 4h). After drying, the fibers spontaneously formed entangled bundles with random arrangements of blue, green, and reddish‐white strands, as shown in Figure 4i. The fibers demonstrated robust flexibility, allowing for manual manipulation without fracture under the formation of a tight, defined loop, as illustrated in Figure 4j. Moreover, the fibers could be neatly organized and stored by uniformly winding them around cylindrical spools (or bobbins), facilitating convenient handling and scalable packaging of color‐coded radiative cooling threads (Figure 4k). Notably, the wound fibers maintained uniform coloration along their entire length regardless of view angles, further supporting the formation of photonic glass structures within the fibers.

**FIGURE 5:**
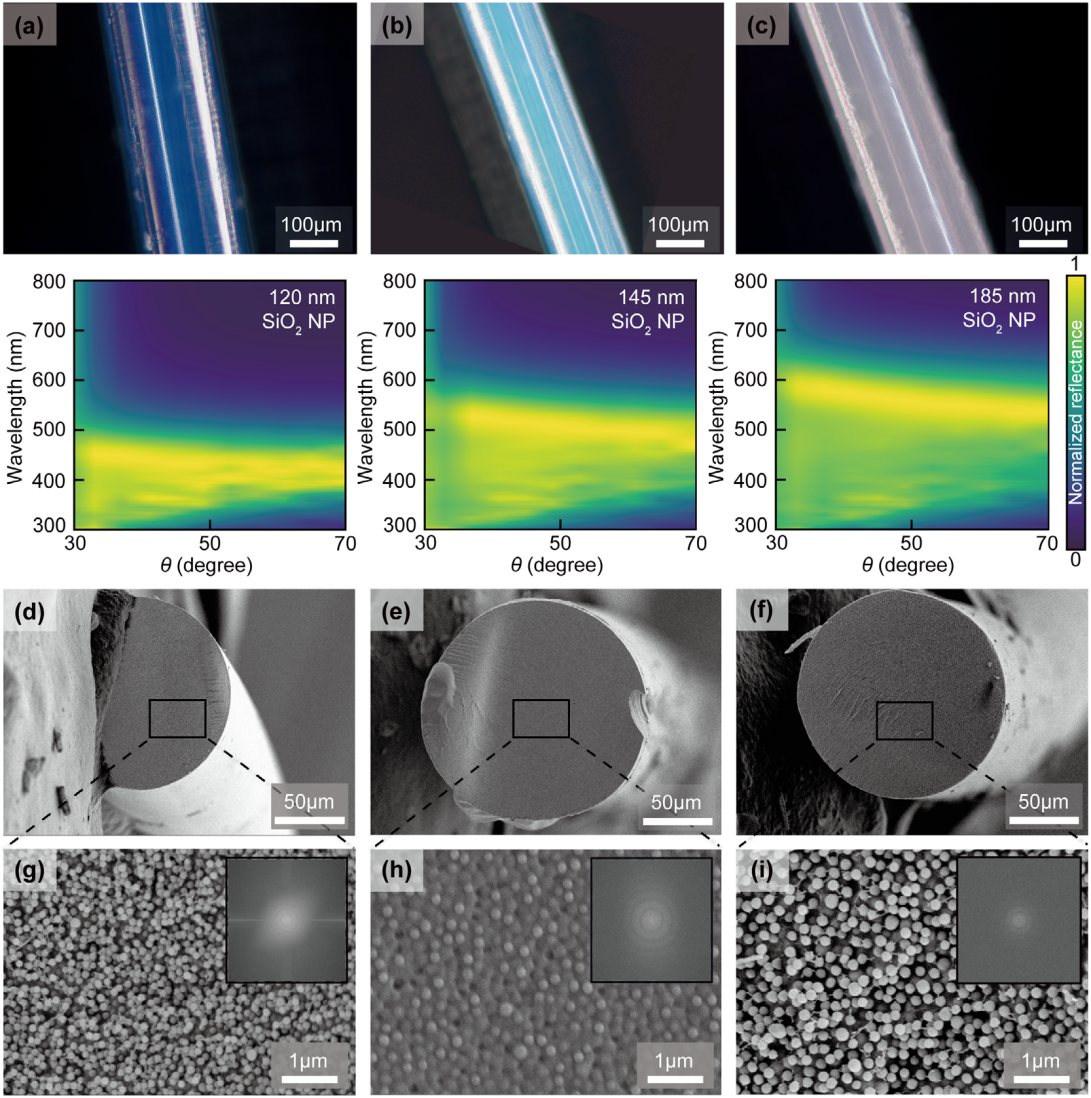
(a)–(c) Dark‐field OM images (top panel) of individual blue (a), green (b), and reddish‐white (c) composite fibers (numerical aperture = 0.3; incident angle = 0°), showing uniform color appearance along the entire fiber surfaces without iridescence or angular hue variation, confirming the angle‐independent structural coloration. Experimentally measured angle‐resolved reflectance spectrum of their SiO_2_ NP–ETPTA composite films (bottom panel). The incident light angle was fixed at 13°, while the detection angle (*θ*) was varied. (d)–(f) Cross‐sectional SEM images of the corresponding blue, green, and reddish‐white fibers, indicating homogeneous internal morphology and uniform NP distribution throughout the fiber cores. (g)–(i) High‐magnification surface SEM images and their corresponding fast Fourier transform (FFT) insets, revealing randomly packed SiO_2_ NPs with no long‐range periodicity, characteristic of amorphous photonic glass structures responsible for the observed noniridescent reflection.

To examine this angle‐independent colorization in more detail, we observed individual fiber strands under dark‐field OM, as shown in Figure 5a–c (numerical aperture [NA] = 0.3; incident angle = 0°). The blue (Figure 5a), green (Figure 5b), and reddish‐white (Figure 5c) fibers exhibited spatially uniform coloration across their entire surfaces without iridescent shifts (top panels of Figure 5a–c). This behavior confirms the angle‐independent structural coloration characteristic of photonic glasses, consistent with observations of planar photonic glass films (bottom panels of Figure 5a–c). To confirm the structural origin of the coloration, we performed scanning electron microscopy (SEM) analysis of the fiber surfaces (Figure 5d–i). The images reveal that the SiO_2_ NPs are arranged in a disordered, random fashion, with no evidence of long‐range periodicity, which was consistent with the formation of amorphous photonic glass structures responsible for the observed noniridescent reflection. The minor variations in surface morphology among SEM images can be attributed to artifacts such as the fracture surface created during sample preparation or local charging during imaging. The spatially uniform coloration observed in dark‐field OM confirms that the internal microstructure remains consistent throughout the entire fiber.

### 2.4 Photonic and Cooling Properties of Assembled Fibers

**FIGURE 6:**
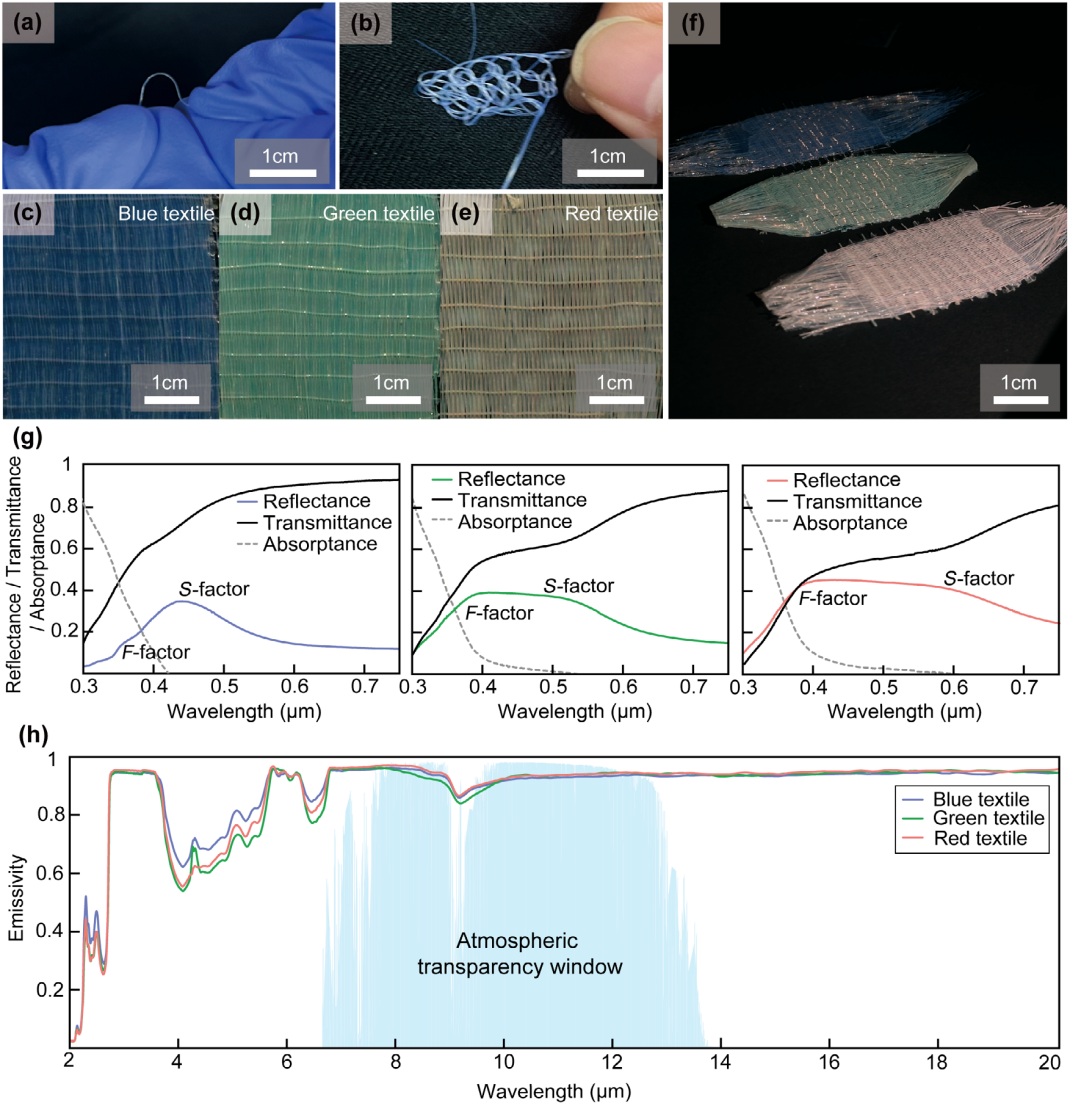
(a), (b) Photographs demonstrating the flexibility of individual photonic glass fibers. (c)–(f) Hand‐woven lattice‐type fabrics assembled from the fibers, mimicking the crossed‐strip pattern of an apple‐pie crust, showing uniform weave structure with an areal (open) fraction below ≈10%. Blue, green, and reddish‐white fabrics prepared from 120, 150, and 180 nm SiO_2_‐based fibers, respectively, exhibiting and uniform coloration regardless of bending or viewing angle. (g) Optical spectra of the woven textiles measured using an integrating‐sphere‐equipped UV–Vis spectrophotometer, displaying characteristic form‐factor (F‐factor) and structure‐factor (S‐factor) scattering features of photonic glasses, consistent with theoretical predictions. (h) Measured hemispherical emissivity spectra of blue, green, and reddish‐white SiO_2_–ETPTA photonic glass textiles, showing high and broadband mid‐IR emissivity (>0.9) across the atmospheric transparency window.

Because our photonic glass fiber is bendable and elastically recoverable (Figure 6a,b), it can be hand‐woven into textiles. For simplicity, we fabricated a lattice weave, resembling the crossed strips of an apple‐pie crust by hand (Figure 6c–f). The resulting fabric has an open‐area (areal) fraction less than ∼10%; a quantitative analysis of the areal fraction is provided in Figure S13. The woven fabric remained flexible, and we routinely produced ∼4 × 4 cm pieces irrespective of fiber coloration (Figure 6f).

As expected from theory (Figure 2), the woven fabric exhibited the spectral hallmarks of photonic glasses, with contributions from both form‐factor and structure‐factor scattering, which were characterized using an integrating‐sphere‐equipped UV–Vis spectrophotometer (Figure 6g). In these measurements, scattering manifested as enhanced total hemispherical reflectance accompanied by reduced transmittance. The form factor feature occurred at shorter wavelengths (350–390 nm); however, because ETPTA strongly absorbs in this range, this feature contributed little to the observed transmission loss. By contrast, at the longer wavelengths (>400 nm) where the absorptance is negligible, structure factor resonances in the B, G, and R bands increased reflectance and correspondingly decreased transmittance, with the structure‐factor peak broadening continuously from B to G to R, consistent with prior results [42, 43, 55, 56, 57, 58]. Consequently, the integrated solar reflectance increased in the order B < G < R, a trend advantageous for daytime radiative cooling.

It is noteworthy that the current single‐layer array serves as a representative configuration to characterize the photonic properties of the glass fibers rather than an optimized apparel textile. This hand‐woven lattice exhibits a nonnegligible visible transmittance under ∼10% of open‐area fraction. For practical clothing applications, opacity can be enhanced by increasing the fiber areal density, adopting denser weave patterns, or employing multilayer stacking. Such structural adjustments involve a design trade‐space where transparency must be balanced with color saturation and solar reflectance, arising from isotropic structural coloration, as well as breathability to meet the requirements of functional radiative cooling textiles.

In contrast to the visible regime, the fiber microstructure is deeply subwavelength in the mid‐IR, so the textiles behave as an effective homogeneous medium. Accordingly, the RGB colloidal photonic textiles exhibited nearly identical hemispherical absorptivity/emissivity (α ≈ ε) exceeding 0.9, independent of their visible‐band solar reflectance (Figure 6h). Because the SiO_2_/ETPTA composite acts as a broadband thermal emitter, this high ε is maintained across the whole mid‐IR atmospheric window. Such broadband emission is advantageous over selective emitters for cooling substrates that are significantly hotter than ambient, as it couples efficiently to the wide blackbody spectrum of the hot surface [1, 59, 60, 61].

**FIGURE 7:**
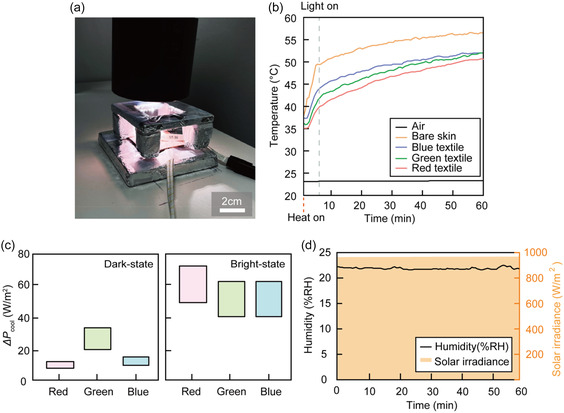
Indoor solar‐simulator test for evaluating textile temperature on a skin simulator. (a) Photograph of the experimental setup for the indoor solar‐simulator measurement, in which textile samples were placed on top of a skin‐mimicking simulator and illuminated under controlled conditions. (b) Time‐dependent surface temperature profiles of bare skin and textile‐covered samples measured during solar irradiation. The dashed line indicates the moment when the solar simulator was turned on. (c) Summary of the additional cooling power relative to the bare‐skin baseline ($\Delta P_{cool}$) for the blue (B), green (G), and reddish‐white (R) textiles under dark‐ and bright‐state conditions. (d) Solar irradiance and ambient humidity recorded throughout the experiment to confirm stable illumination and environmental conditions.

To evaluate the thermal performance of the RGB photonic‐glass fabric under controlled yet physiologically relevant conditions, an indoor solar‐simulator experiment was conducted using a heated skin‐mimicking substrate (Figure 7a). The bare skin phantom serves as a reproducible baseline to quantify how the textile layer modifies the net heat load on skin under solar illumination. We note that real human skin is highly emissive (*ε* ≈ 0.98), whereas the silicone‐based skin phantom used here exhibits *ε* ≈ 0.8–0.9 (Figure S14). Under 1 sun irradiation, bare skin/phantoms also absorb a nonnegligible fraction of solar radiation, particularly at short wavelengths (<600 nm) [62]. The design goal of our photonic‐glass textile is therefore to reduce solar heat gain via strong visible/NIR scattering and reflection while maintaining high mid‐infrared emissivity (>0.9), so that metabolic heat dissipation is not impeded. A systematic benchmark against specific commercial dyed fabrics (which vary widely by weave, fiber composition, and coloration) is beyond the scope of this work and is an important direction for future studies.

The woven fabric is highly flexible and can conformally wrap curved surfaces such as skin, suggesting use as a daytime radiative‐cooling patch/bandage [63, 64, 65, 66]. The textile samples were placed in direct contact with an artificial skin simulator that reproduces both the optical (reflectance/transmittance in the visible–near‐infrared region and high mid‐infrared emissivity) and thermal (basal metabolic heat flux) characteristics of human skin (see Figure S14). A silicone rubber heater beneath the skin surface supplied a constant heat flux of 104 W m^−2^, corresponding to the average metabolic heat generation of the human body, while the surface temperature was stabilized near 36°C prior to illumination.

The experimental setup was illuminated using an AM 1.5G solar simulator operating at an irradiance close to 1 sun. To mitigate uncontrolled forced convection from incidental air currents (e.g., HVAC drafts) while minimally perturbing radiative heat transfer, a thin low‐density polyethylene (LDPE) film (transparent in the visible and mid‐infrared spectral ranges) was mounted above the samples as a draft shield. We emphasize that this film does not eliminate natural convection; rather, it improves reproducibility by stabilizing the local airflow boundary condition. All samples were tested under identical configurations, and no forced airflow or fan was applied, such that heat exchange at the surface was governed primarily by natural convection and thermal radiation.

The temperature of the simulated skin was monitored for 30 min with and without solar illumination and compared with a bare‐skin baseline, as detailed in Figure 7b. When irradiated under 1 sun, the photonic‐glass textile suppresses solar‐induced heating and reduces the steady‐state skin temperature by 4°C–5°C, demonstrating passive temperature reduction relative to the bare‐skin baseline. Even in the dark (solar simulator off), the textile‐covered sample maintains a lower steady‐state temperature than the bare skin phantom. This is consistent with the high mid‐infrared emissivity of the photonic‐glass textiles (Figure 6h), which is comparable to or higher than that of the skin phantom used here (Figure S14). The absolute temperatures of all samples gradually increase in the dark due to radiative heat exchange with the warmer surrounding lab environment, although the constant simulated metabolic heat flux is continuously maintained throughout the experiment. This observation indicates that the textile does not hinder radiative heat dissipation and can reduce net heat gain under both illuminated and nonilluminated indoor conditions.

Upon solar illumination, all samples exhibit an increase in surface temperature due to solar heating. Nevertheless, the RGB photonic‐glass textiles consistently maintain significantly lower skin temperatures than the bare‐skin control throughout the measurement, with temperature reductions of approximately 4°C–5°C under steady‐state irradiation (Figure 7b). This temperature suppression indicates a substantial reduction in the net solar heat load delivered to the skin, arising from strong scattering and reflection of visible and near‐infrared radiation by the photonic‐glass fibers, while simultaneously preserving broadband mid‐infrared emissivity for efficient thermal radiation. Consistent with the optical characterization shown in Figure 6g, the solar reflectance increases in the order of R, G, and B textiles (with the opposite trend observed for solar transmittance), leading to a corresponding hierarchy in skin temperature under solar‐simulator illumination. As a result, the skin temperature remains lowest for the R textile, followed by the green and blue textiles, throughout the illumination period. At t = 60 min, the corresponding additional cooling power relative to the bare‐skin baseline was estimated to be $\Delta P_{cool}$ ≈ 41–62 W m^−2^ for the blue textile, 42–62 W m^−2^ for the green textile, and 49–73 W m^−2^ for the reddish‐white textile (see Supporting Information for details of the $\Delta P_{cool}$ estimation). The obtained $\Delta P_{cool}$ for dark‐ and bright‐state are summarized in Figure 7c. Throughout the experiment, the solar irradiance was maintained at an average value of approximately 980 W m^−2^, and the ambient relative humidity remained stable near 23% RH (Figure 7d), ensuring well‐controlled environmental conditions. Together, these results demonstrate that the photonic‐glass textiles provide effective thermal regulation on a skin‐mimicking substrate under solar illumination, while retaining high mid‐infrared emissivity, highlighting their potential for practical wearable radiative‐cooling applications.

**FIGURE 8:**
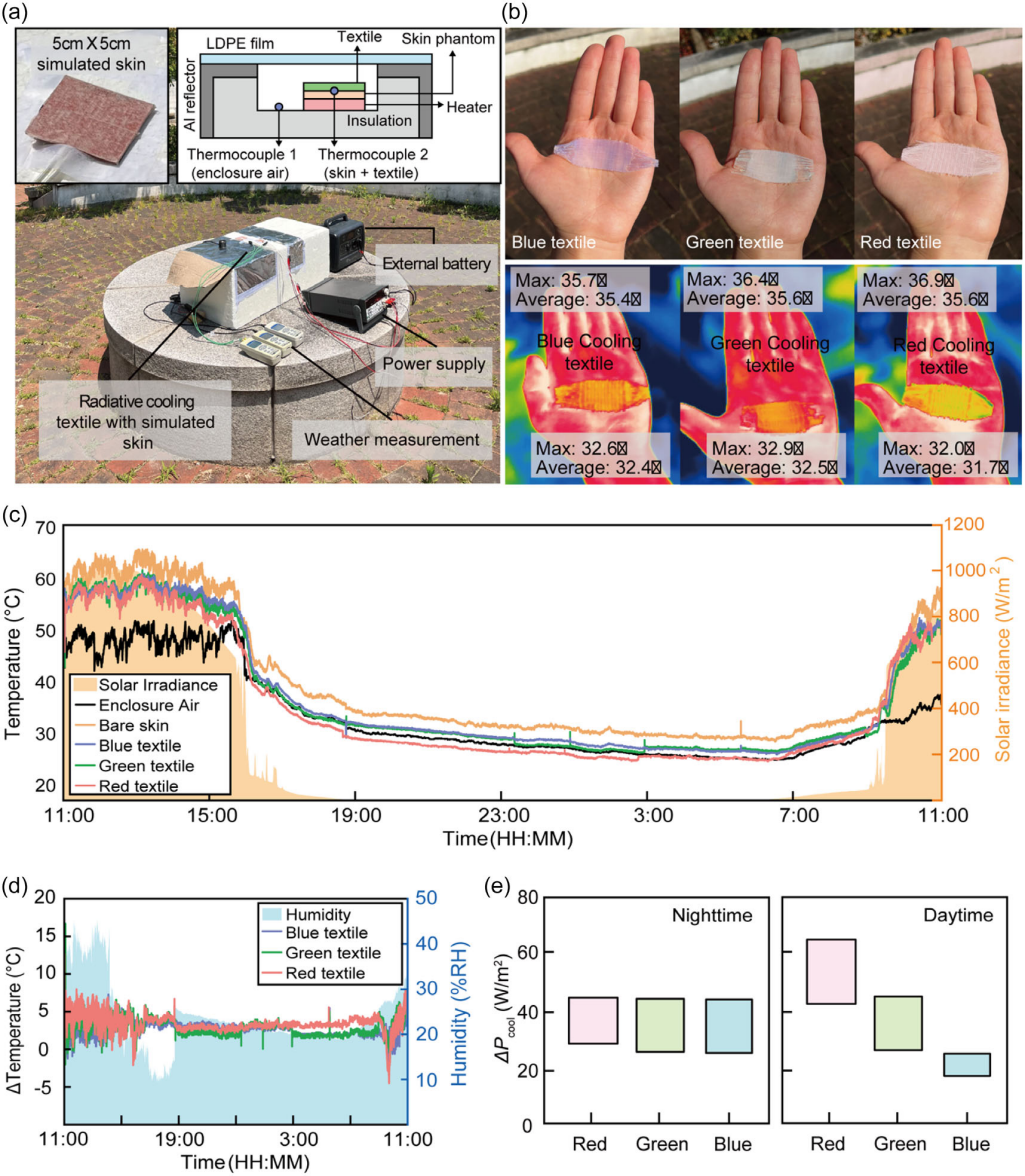
Outdoor radiative cooling performance of photonic‐glass textiles. (a) Photograph of the outdoor experimental setup, where the textiles were placed on skin phantoms and exposed to natural solar illumination while temperatures were monitored continuously. (b) Infrared thermography images of a human palm covered with the B, G, and R photonic‐glass textiles under direct sunlight. The reddish‐white textile exhibits the strongest cooling effect. (c) Outdoor 24 h temperature depression (ΔT) measurements for each textile relative to the bare‐skin phantom, illustrating superior daytime cooling for the reddish‐white textile. (d) Outdoor 24 h temperature depression (ΔT) and relative humidity measurements, demonstrating nighttime cooling for all samples (with minor color‐dependent variations) and superior daytime cooling for the reddish‐white textile. (e) Extracted $\Delta P_{cool}$ for the three textiles during daytime (11:00–15:00) and nighttime (23:00–03:00) periods. The reddish‐white textile exhibits the highest cooling power during the daytime due to its highest solar reflectance, while all samples show comparable ranges during the nighttime due to their similar mid‐IR emissivity.

Outdoor tests were conducted in front of the KU R&D Center, Korea University, Seoul, 37°35′24.0″N 127°01′36.4″E) (GPS coordinates provided in the Methods) on 10 September 2025, spanning both daytime and nighttime conditions (Figure 8a). Each 5 × 5 cm photonic‐glass textile was mounted on a skin‐mimicking phantom calibrated to reproduce the basal radiative heat flux of human skin and monitored under natural solar illumination and local environmental conditions, as mentioned above. For clarity, the air temperature recorded in our setup corresponds to the local air temperature inside the wind‐shielded enclosure that surrounds the samples (inset, Figure 8a); the meteorological ambient air temperature outside the enclosure on the test day (Seoul) is provided in Supporting Information (Section 11). Qualitative demonstrations on a human palm under direct sunlight already reveal distinct thermal responses among the three textiles: IR thermography shows that the reddish‐white textile yields the lowest surface temperature, followed by the green and blue textiles (Figure 8b). This trend is reproduced quantitatively in the outdoor skin‐phantom measurements. During daytime operation under strong solar irradiance, the temperature depression of the simulated skin follows the order B < G < R (Figure 8c), indicating progressively stronger cooling from the blue to the reddish‐white textile. This hierarchy directly reflects the solar‐band optical properties of the textiles. As shown previously (Figure 6g), solar reflectance increases systematically from the B to the G and R photonic‐glass textiles. Because the measured solar absorptance above 400 nm is low (Figure 6g), the increased reflection nonetheless reduces the net solar heat load at the skin surface, even though the current textiles are semi‐transparent due to the open‐area weave. Time‐averaged analysis over the daytime window (11:00–15:00) yields ΔP values of approximately 16–25 W m^−2^ for the blue textile, 28–42 W m^−2^ for the green textile, and 44–65 W m^−2^ for the reddish‐white textile (Figure 8e; see detailed estimation process summarized in Supporting Information), confirming that enhanced solar scattering and reflection dominate daytime cooling performance.

In contrast, nighttime measurements (23:00–03:00), when solar input is absent, show that all three textiles maintain cooling relative to the bare‐skin phantom, with broadly comparable magnitudes (Figure 8d). The extracted nighttime $\Delta P_{cool}$ values are 28–42 W m^−2^ for the B and G textiles and 30–46 W m^−2^ for the R textile (Figure 8e). While the overall nighttime performance is primarily governed by the high and broadband mid‐infrared emissivity shared by all textiles (Figure 6h), small differences among colors can arise from variations in textile openness/thickness (affecting nonradiative heat transfer) and slight emissivity variations (see Supporting Information, Section 11). Notably, the bare‐skin phantom maintains a higher steady‐state temperature than the textile‐covered samples even in the absence of solar input. This is attributed to the fact that the emissivity of skin phantom (0.8–0.9, Figure S14) is lower than that of the textiles (>0.9). Under a constant metabolic heat flux, the higher effective emissivity of the textile‐covered system allows for more efficient heat radiation, resulting in a lower steady‐state temperature compared to the bare phantom.

Importantly, these outdoor observations are in excellent agreement with the indoor solar‐simulator measurements (Figure 7), which showed the same ordering of daytime cooling governed by solar‐band scattering and comparable nighttime cooling dictated by mid‐infrared emissivity. The consistency between controlled indoor experiments and 24 h outdoor field tests highlights the reliability of the experimental methodology and the predictive nature of the optical–thermal design principles underlying the photonic‐glass textiles.

Taken together, the outdoor results establish a clear and quantitative causality linking optical design to thermal performance: solar‐band reflectance determines the magnitude of daytime cooling, whereas color‐independent mid‐infrared emissivity governs nighttime radiative cooling. This strong agreement across indoor and outdoor conditions highlights the robustness of the photonic‐glass textile platform for practical, real‐world radiative‐cooling applications.

## 3 Conclusion

We have demonstrated a photonic materials platform that unites esthetic versatility and thermal functionality through the integration of amorphous photonic glass optics and radiative cooling physics. By exploiting the entropy‐driven self‐assembly of SiO_2_ colloids within a photocurable ETPTA matrix and solidifying them via microfluidic extrusion, we achieved elastic fibers exhibiting vivid, angle‐independent structural coloration and broadband mid‐IR emissivity. The dual‐band optical response, such as solar reflection in the visible and strong phonon‐mediated emission in the mid‐IR enables passive heat rejection from hot surfaces without external power. Importantly, unlike conventional dye‐based colored radiative‐cooling fibers, whose hues inevitably fade or bleach under prolonged sunlight exposure, the colorization in our fibers originates from structural photonic scattering rather than molecular absorption. This structural coloration provides permanent, lightfast color stability suitable for long‐term outdoor applications, even under intense UV irradiation. Moreover, fiber weaving enhances diffuse scattering and scalability, establishing a pathway toward color‐tunable, flexible, and manufacturable cooling textiles. This strategy expands the design space of radiative cooling materials beyond conventional whitish or pigment‐based systems, offering a foundation for next‐generation energy‐saving fabrics applicable to architecture, personal thermal comfort, and wearable photonic systems.

## 4 Experimental Section

### 4.1 Synthesis of Silica Colloids

All chemical reagents, including tetraethyl orthosilicate (Aldrich, ≥99.0%), ammonia solution (Aldrich, 25%), and anhydrous ethanol (Aldrich, ≥99.5%), were used as received. Deionized (DI) water (Milli‐Q water) was used as the solvent for the synthesis of silica colloids. We synthesized silica colloids in a one‐step process using the solvent varying method, which was modified from the Stöber method. Given the mixture of TEOS, ammonia solution, and DI water (6, 8, and 3 mL, respectively), we varied the volume of ethanol from 70 to 90 mL. Through this, the size of silica colloids was precisely tuned from 120 to 180 nm. The reaction temperature was 60°C, and the mixture was stirred for 2 h. The synthesized silica colloids were centrifuged at 1000 rcf for 30 min and washed two times with ethanol. After ethanol was completely evaporated, the silica colloids were redispersed in pure ethanol.

### 4.2 Fabrication of Microfluidic Devices

The master mold for the microfluidic device was fabricated using standard soft‐lithography procedures. A 4‐inch prime silicon wafer served as the substrate. SU‐8 50 (MicroChem) was spin‐coated at 500 rpm for 10 s and 500 rpm for 60 s, followed by prebake at 65°C for 20 min and soft bake at 95°C for 1 h 20 min. UV exposure was carried out on a mask aligner (MDA‐400N, Midas System) at 30 mW cm^−2^ for 32 s through the photomask onto the flat SU‐8 layer. Postexposure bake was performed stepwise at 65°C for 1 min and 95°C for 40 min. The patterns were developed using the manufacturer's SU‐8 developer (MicroChem), then rinsed with isopropyl alcohol (IPA, Sigma–Aldrich) and deionized (DI) water, yielding the master mold.

PDMS prepolymer was prepared at a 10:1 (base curing agent) ratio, degassed under vacuum, and gently poured over the master. Curing at 85°C for 1 h produced the elastomer, which was then peeled from the mold. The replica was sequentially rinsed with triethylamine solution, ethyl acetate, and acetone and dried 6 h at 65°C. Immediately before assembly, the PDMS and a glass slide were treated by oxygen plasma (48 W, 1 min, 20 sccm, 0.09 Torr) and brought into conformal contact to form an irreversible bond.

### 4.3 Fabrication of Hand‐Woven Photonic Glass Textiles

SiO_2_ NP–ETPTA fibers produced by the microfluidic process were thermally relaxed on a flat substrate to remove residual curvature. The straightened fibers were subsequently hand‐woven on a jig into a lattice (‘apple‐pie’) pattern with orthogonal crossings and uniform spacing. The woven mat was secured along the perimeter by fixing the fiber ends (mechanical clamping or spot bonding), released from the jig, and trimmed to the specified outline. The textile was then formed to the target dimensions, yielding a hand‐woven photonic glass sheet used as a radiative cooling textile.

### 4.4 Operation of Microfluidic Device

Aqueous 10 wt% poly vinyl acetate (PVA, Sigma–Aldrich) solution was used as the dispersed phase, and SiO_2_ NP–ETPTA (20vol% of SiO_2_) served as the continuous phase. The two fluids were delivered to separate inlets of the T‐junction microfluidic device via pneumatic pressure control (FLOW EZ 2000) through PEEK tubing (natural, 1/16″ OD × 0.040″ ID). Operating pressures were set to 300 mbar (dispersed) and 180 mbar (continuous). The T‐ junction microfluidic device was wrapped with a UV‐opaque cover, leaving only the outlet exposed. At the outlet, extruded SiO_2_ NP–ETPTA fibers were irradiated with 365 nm wavelength of light to induce in situ curing and were continuously drawn into a deionized (DI) water bath. The fibers collected in the bath were washed three times with DI water and dried at room temperature to obtain free‐standing SiO_2_ NP–ETPTA fibers.

### 4.5 Optical Characterization of R, G, B Colored Radiative Cooling Textiles

The optical properties of the R, G, B radiative cooling textiles were characterized using a Fourier‐transform IR (FT‐IR) spectrometer (INVENIO‐X, Bruker) equipped with appropriate integrating sphere accessories. A PTFE‐coated integrating sphere was used for measurements in the visible and near‐IR range (400–900 nm), while a gold‐coated integrating sphere was employed for the mid‐IR range. All measurements were performed under normal incidence, with the incident light directed from the air side onto the ETPTA surface, followed by the opaline composite layer. The reflectance and transmittance spectra were collected using the OPUS software, and absorptance was calculated by subtracting the measured reflectance and transmittance from unity. To ensure a consistent and fair comparison, all textile samples were prepared with the same total thickness of 400 μm.

### 4.6 Setup for Measuring Angle‐Independence of the R, G, B Colored Radiative Cooling Textiles

The angle‐resolved reflection spectra were quantified by using FTIR spectroscopy (INVENIO‐X) equipped with an angle adjustment accessory. The incident light angle was fixed at 13°, while the detection angle (*θ*) was varied from 30° to 69°, with 1° intervals.

### 4.7 Setup for Outdoor Radiative Cooling Performance Tests

Daytime radiative‐cooling measurements were performed at the front of the KU R&D Center, Korea University, Seoul, Republic of Korea (37°35′24.0″N, 127°01′36.4″E). The custom setup comprised of a Styrofoam base, a thermometer (TM‐947SD), a K‐type thermocouple (ST‐50), a solar power meter (SPM‐1116SD), a hygrometer (AM‐4237SD), flexible heaters (Omega KHLVA‐101/1‐P), a programable power supply (Keithley 2230‐30−1), and a laptop (LG gram). Thermocouples were positioned at the interface between the flexible heater and the sample, and the samples were mounted on a Styrofoam support inside the enclosure (right inset image in Figure 8a).

### 4.8 Monte Carlo Simulation

To theoretically quantify the optical transport properties of photonic glass fibers, a three dimensional Monte Carlo simulation code was developed based on the previously reported photon packet Monte Carlo approach [44]. The custom python code used for this Monte Carlo simulation is provided in GitHub (https://github.com/NEOlab‐code/Monte‐Carlo‐simulation‐for‐photonic‐glass‐fiber.git).

## Supporting Information

Additional supporting information can be found online in the Supporting Information section.

## Author Contributions

S.L. conceived original idea and supervised project. S.A. synthesized silica colloids/fibers/textiles, developed microfluidic chip, and conducted photonic characterization and radiative cooling experiments. J.L. theoretically analyzed photonic glass. S.K. contributed to silica synthesis and segment analysis. E.I. contributed photonic glass analysis. Y.D.C. contributed to radiative cooling analysis. H.H.K. contributed the development of structural fluids. H.L. and S.L. supervised the project. S.L., S.A., J.L. wrote the manuscript with the feedback from all authors.

## Funding

This study was supported by National Research Foundation (NRF) of Korea (RS‐2022‐NR068141), the KU‐KIST research program (2V09840‐23‐P023) and a Korea University grant.

## Conflicts of Interest

The authors declare no conflicts of interest.

## Supporting information

Supporting information is avaliable from the Wiley Online Library or from the author.

## Data Availability

The data supporting the plots and other findings of this study are available from the corresponding authors upon reasonable request. The code for the Monte Carlo simulationused in this work is available in the following GitHub repository: https://github.com/NEOlab‐code/Monte‐Carlo‐simulation‐for‐photonic‐glass‐fiber.git.
