# Supplementary material for: Colloidal Photonic Fibers for Reflectively Colorful Radiative Cooling Fabrics
Source: Small Sci. 2026 Apr 21;6(4):e70273. doi: 10.1002/smsc.70273 (PMC13099592; doi:10.1002/smsc.70273)
Supplement: Supplementary file 1 — Supporting information is avaliable from the Wiley Online Library or from the author. [file SMSC-6-e70273-s001.zip › smsc70273-sup-0001-SuppData-S1/smsc70273-sup-0001-SuppData-S1.pdf]

Supporting Information for

Colloidal photonic fibers for reflectively colorful radiative cooling fabrics

Sewon Ahn[1,+], *Jaewon Lee*[1,+], *Soyul Kwak*[1], *Eunji Im*[2], *YongDeok Cho*[1], *Hyeon Ho Kim*[1], *Heon Lee*,[3,*] and *Seungwoo Lee*[1,2,4,5*]

[1]KU-KIST Graduate School of Converging Science and Technology, Korea University, Seoul 02841, Republic of Korea

[2]Department of Biomicrosystem Technology, Korea University, Seoul 02841, Republic of Korea

[3]Department of Materials Science and Engineering, Korea University, Seoul 02841, Republic of Korea

[4]Department of Integrative Energy Engineering, Korea University, Seoul 02841, Republic of Korea

[5]Center for Opto-Electronic Materials and Devices, Post-Silicon Semiconductor Institute, Korea Institute of Science and Technology (KIST), Seoul 02792, Republic of Korea

[*]Email: seungwoo@korea.ac.kr; heonlee@korea.ac.kr

[+]Equally contributed to this work

Keywords: radiative cooling, metamerism, colloids, structured fluids, photonic glass

Contents:

1. The intermolecular force between silica nanoparticles (NPs) in ETPTA
2. Analytical and numerical analyses of silica photonic glasses
    2.1. Detailed method for modeling and full wave calculation of the disordered colloidal photonic glass structure
    2.2. Born type approximation based analysis of photonic glasses
3. Light propagation in SiO_2 NP-ETPTA composite
4. Material property of SiO_2 NP-ETPTA composite in mid-infrared (IR) range
5. Monte Carlo simulation model for the analysis of photonic fiber
6. Synthesis of monodisperse silica NPs
7. Microfluidic chip
8. Areal factor of assembled textile
9. Details of simulated skin
10. Indoor radiative cooling performance
11. Details of outdoor cooling power calculations

1. The intermolecular force between silica nanoparticles (NPs) in ETPTA

The intermolecular forces between two colloidal bodies are commonly discussed within the Derjaguin–Landau–Verwey–Overbeek (DLVO) framework, where the attractive contribution is dominated by van der Waals (vdW) interactions. For two identical spherical particles of radius R separated by a surface-to-surface gap H, the vdW interaction energy can be approximated as[1] :

$$V_{vdW} = -\frac{A_H \times R}{12H} \qquad \text{(Equation S1)}$$

Here, A_H is the effective Hamaker constant for the silica–medium–silica configuration, which reflects the material polarization response across the medium. In aqueous media, silica–silica interactions are typically characterized by Hamaker constants by $6.5 * 10^{-21} J$. In contrast, in ETPTA the vdW attraction is expected to be strongly suppressed because ETPTA is nearly refractive-index matched to silica (silica n≈1.46, ETPTA =1.471). Following the Tabor–Winterton (TW) approximation eq S2, the dispersive contribution to the Hamaker constant can be estimated primarily from the n of the interacting bodies and the medium, and is therefore markedly reduced under n-matching conditions. Using this n scaling, we estimate

$$A_H(\text{ETPTA}) \approx 0.01 * A_H(water) \sim 6.5 \times 10^{-23} J$$

For identical bodies across a medium, the TW scaling for the dispersive contribution can be written as[2]

$$A_H \propto \frac{(n_{sio_2}^2 - n_{sio_2}^2)^2}{n_{sio_2}^2 + n_m^2} \qquad \text{(Equation S2)}$$

where, n_m is the refractive index of the medium. The resulting vdW attraction for silica dispersed in ETPTA is therefore expected to be negligible compared with that in water. This comparison is summarized in **Figure S1**, which contrasts the vdW attraction for silica in water and ETPTA.

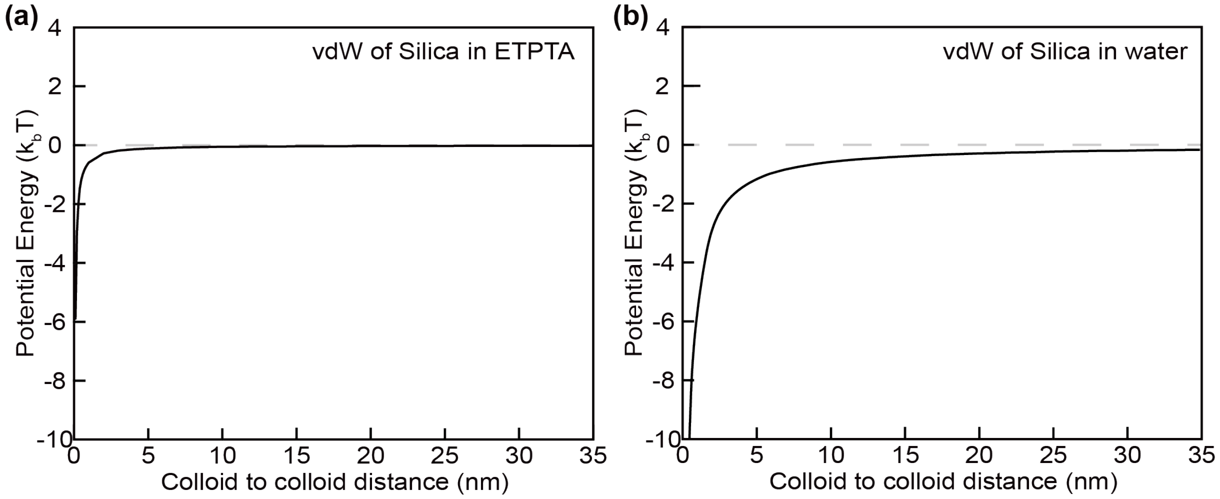

Figure S1. Comparison of vdW interaction potential of silica in ETPTA (a) and water (b).

2. Analytical and numerical analyses of silica photonic glasses

2.1. Detailed method for modeling and full wave calculation of the disordered colloidal photonic glass structure

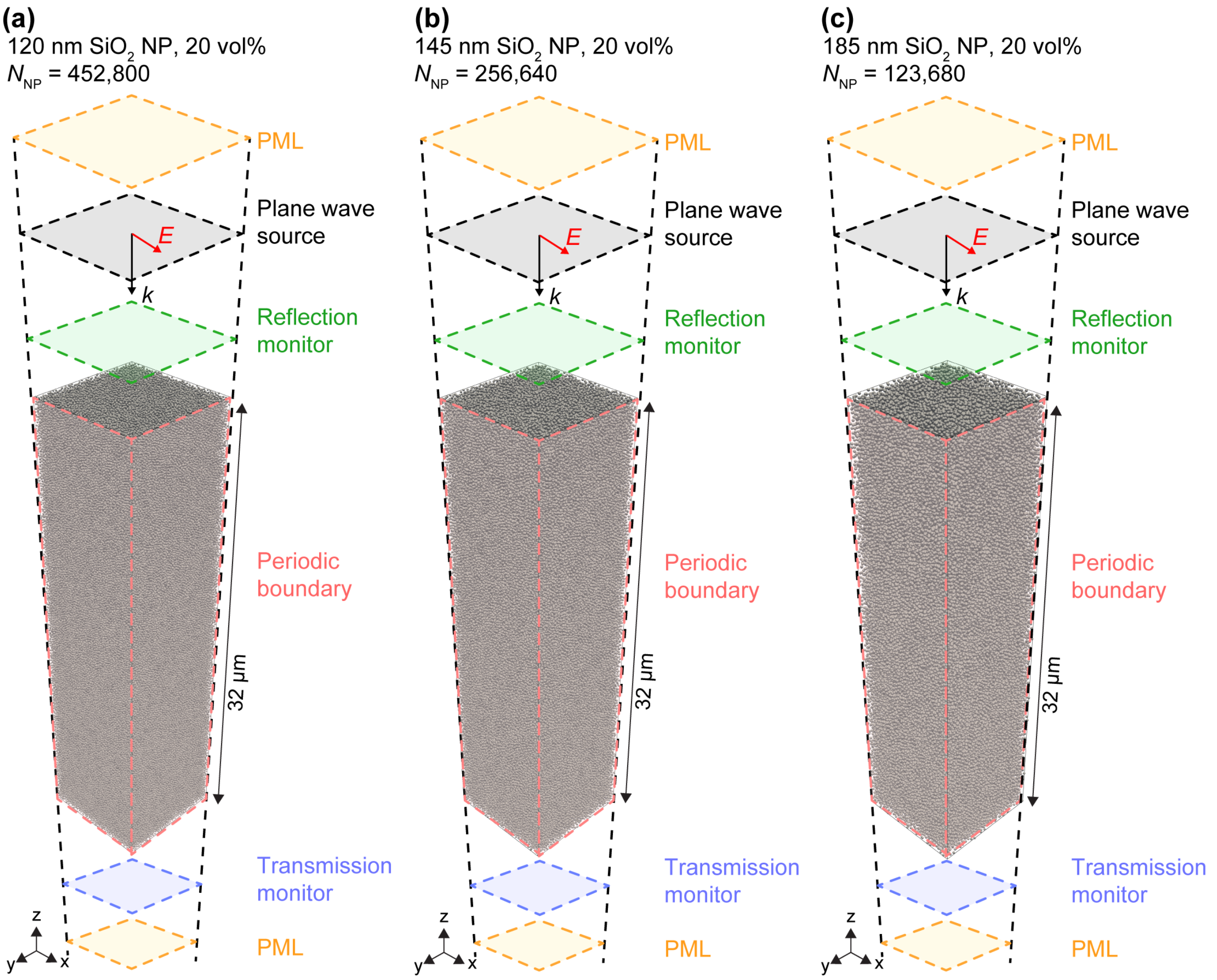

Figure S2. Schematic illustration of the FDTD simulation domains for photonic glasses composed of (a) 120 nm, (b) 145 nm, and (c) 185 nm SiO_2 nanoparticles (NPs). The total number of NPs (N_{NP}) included in each 8 μm × 8 μm × 32 μm simulation volume is indicated. Key simulation components, including the plane wave source, monitors, periodic boundaries, and perfectly matched layers (PML), are also labeled.

The numerical calculation model for the photonic glass (i.e., silica NP dispersion in an ETPTA matrix) was generated using a hard-sphere packing generation algorithm. Specifically, the hard spheres were modeled as composite particles consisting of a silica core and an ETPTA shell to account for the spatial arrangement of SiO_2 NPs governed by short-range repulsive forces. To reproduce the experimental conditions for the blue, green, and red photonic fibers, the silica core diameters were set to 120, 145, and 185 nm, respectively. To strictly maintain a silica NP volume fraction of 20% across all samples, the ETPTA shell thicknesses were adjusted to 26.5, 32.5, and 41 nm, respectively.

The generation of the disordered packing structure involved a two-step process. First, a force-biased algorithm was utilized to generate a randomly packed geometry with an initial volume fraction of approximately 55%. Second, the Lubachevsky–Stillinger algorithm with gradual densification was

employed to further compact the structure, achieving a final volume fraction of approximately 60%, close to the random close packing limit of the colloidal suspension.[3]

For the full-wave electromagnetic analysis, we utilized the open-source finite-difference time-domain (FDTD) software package, Meep.[4] The computational domain was defined with lateral dimensions of 8 μm × 8 μm and a vertical thickness of 32 μm to represent the bulk medium (**Figure S2**). A spatial resolution of 50 pixels/μm was used, yielding 113-414 volumetric voxels per particle depending on the NP diameter. The default MEEP Courant stability factor (0.5) was employed for all simulations. Periodic boundary conditions were applied in the lateral directions (*x* and *y*) to simulate an infinite slab, while a 500 nm thick (corresponding to half of the longest wavelength used in the simulation) perfectly matched layer (PML) was placed at the top and bottom boundaries along the *z*-direction to eliminate reflections of outgoing waves. A 1000 nm of padding region (corresponding to the longest wavelength) was applied in the propagation direction to minimize undesired artifacts. The final simulation models contained 452,800, 256,640, and 123,680 NPs for the 120, 145, and 185 nm SiO_2 NP dispersion, respectively. The NPs were modeled as being fully embedded within the ETPTA matrix, consistent with the experimental SiO_2 NP–ETPTA composite. The refractive index of the polymerized ETPTA and SiO_2 used for the numerical analysis is summarized in **Figure S3**. To calculate the reflectance, the structure was illuminated with an x-polarized broadband plane wave source, and the reflected flux was collected by a monitor positioned above the scattering volume. The simulations were carried out until the electric field amplitude decayed sufficiently to ensure numerical convergence.

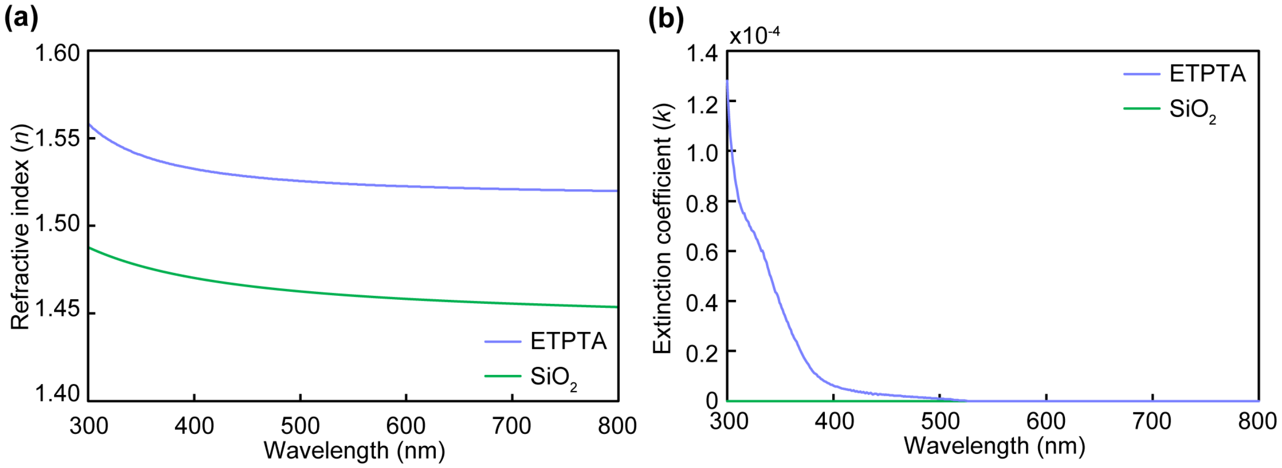

Figure S3. Complex refractive indices of SiO_2 NPs and ETPTA matrix. (a) Refractive index and (b) extinction coefficient.

2.2. Born type approximation-based analysis of photonic glasses

We analyzed the wavelength dependent reflection of photonic glass within the first order (Born type) approximation.[5,6] in which the reflected power is governed by the overlap between the Ewald sphere surface and the squared magnitude of the Fourier transform of the permittivity perturbation $\Delta\varepsilon(\vec{r})$. Following the Ewald construction, the incident wavevector magnitude inside the embedding background is given by $k_{inc} = \frac{\omega}{c} n_b$, and the Ewald sphere is a sphere of radius k_{inc} whose intersections with $\mathcal{F}_T\{\Delta\varepsilon(\vec{r})\}$ define the accessible scattering directions.

In our implementation, we visualize a $k_x - k_y$ section of the reciprocal space intensity and overlay the Ewald circles corresponding to selected wavelengths. To maintain a geometric construction, the radius was evaluated using the real part of the background refractive index. Absorption effects, when present, were treated through the complex permittivity contrast in the motif form factor rather than by a complex Ewald radius.

The photonic glass permittivity distribution was represented as the convolution of a disordered lattice function $l(\vec{r})$ describing the sphere center positions and a motif function $m(\vec{r})$ describing the permittivity perturbation of a single inclusion. Under Fourier transformation, this convolution becomes multiplication, and the ensemble averaged squared magnitude can be expressed as the product of a structure factor $S(\vec{k})$ and a motif form factor $P(\vec{k})$.

$$\langle|\mathcal{F}_T\{\Delta\varepsilon(\vec{r})\}|^2\rangle \propto S(\vec{k}) \cdot P(\vec{k}) \qquad \text{(Equation S3)}$$

For solid spherical inclusions of radius a embedded in a homogeneous background, the motif Fourier amplitude was evaluated analytically as[7]

$$P(k) = \sqrt{\Delta\varepsilon \frac{3[\sin(ka) - (ka)\cos(ka)]}{(ka)^3}} \qquad \text{(Equation S4)}$$

with $\Delta\varepsilon = \varepsilon_s - \varepsilon_b$, where ε_s and ε_b are the sphere and background permittivities, respectively. This expression corresponds to the standard solid sphere motif amplitude used for photonic glass.
Short range order in the photonic glass was modeled by a hard sphere fluid with packing fraction η. The structure factor was evaluated from the Ornstein–Zernike equation with the Percus–Yevick closure, yielding [8,9,10]

$$S(k) = \frac{1}{1 - \rho C(k)}, \qquad \text{(Equation S5)}$$

where ρ is the number density and $C(k)$ is the Fourier transform of the direct correlation function $c(r)$.

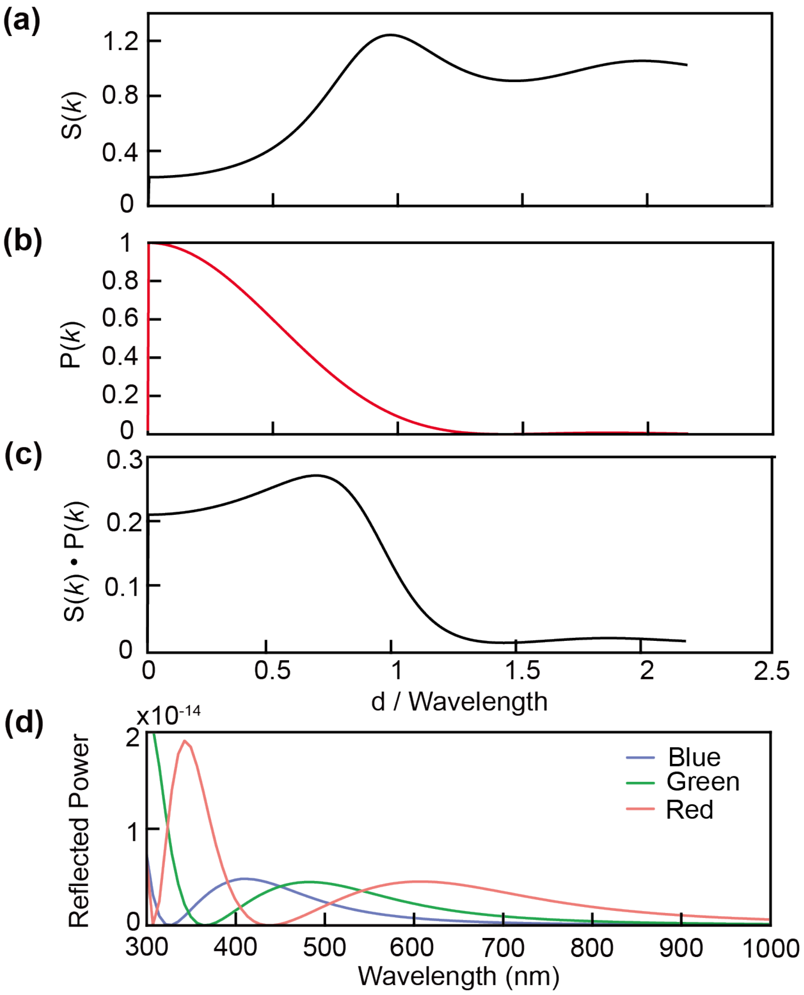

Figure S4. Analytical reproduction of reciprocal space decomposition and reflected power spectra for differently sized SiO_2 NP-based ETPTA photonic glasses. (a) Structure factor $S(k)$ capturing short range positional order. (b) Single particle form factor $P(k)$. (c) Product $(S \cdot P)$, which determines the reciprocal space intensity sampled by the Ewald sphere. (d) Calculated reflected power spectra for NP diameters of d=240 nm (blue), d=290 nm (green), and d=370 nm (red).

3. Light propagation in SiO_2 NP-ETPTA composite

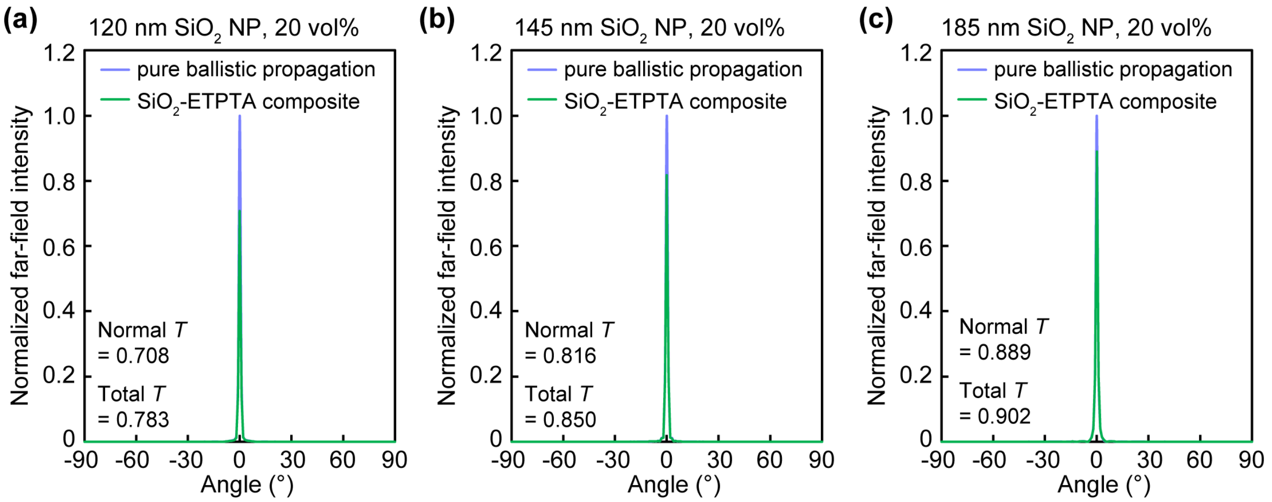

Figure S5. Far-field angular distribution of light transmitted through photonic-glass composites at their respective resonant wavelengths. (a) 120 nm, (b) 145 nm, and (c) 185 nm SiO_2 NP-ETPTA composites. The angular distributions are compared against a pure ballistic propagation reference. The lower normal transmittance value relative to the total transmittance counterpart supports that light propagation through the photonic-glass composite resides in an intermediate regime between pure ballistic transport and diffusive multiple scattering.

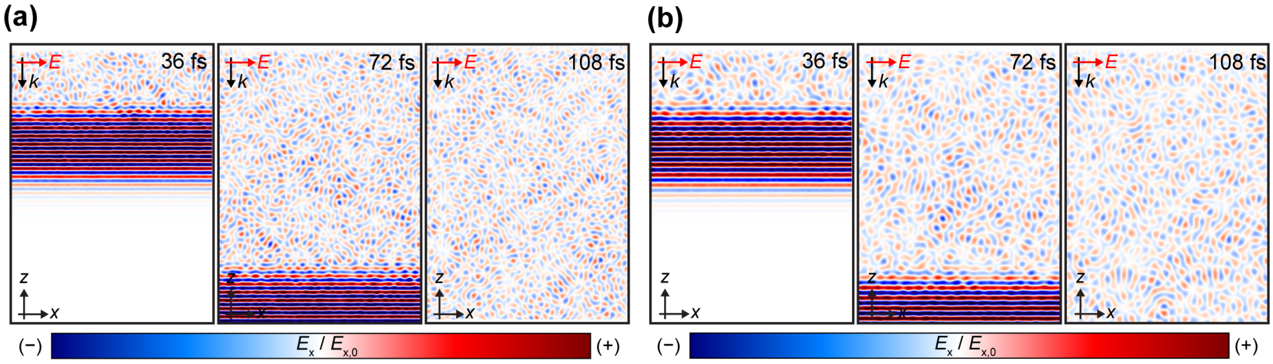

Figure S6. Snapshots of the electric field (E_x) distribution within the photonic glass composites at their respective resonant wavelengths. (a) 145 nm SiO_2 NP-ETPTA composite. (b) 185 nm SiO_2 NP-ETPTA composite. The images display the field evolution at 36, 72, and 108 fs, illustrating light transport in an intermediate regime between ballistic propagation and weak multiple scattering at resonance.

4. Material property of SiO_2 NP-ETPTA composite in mid-infrared (IR) range.

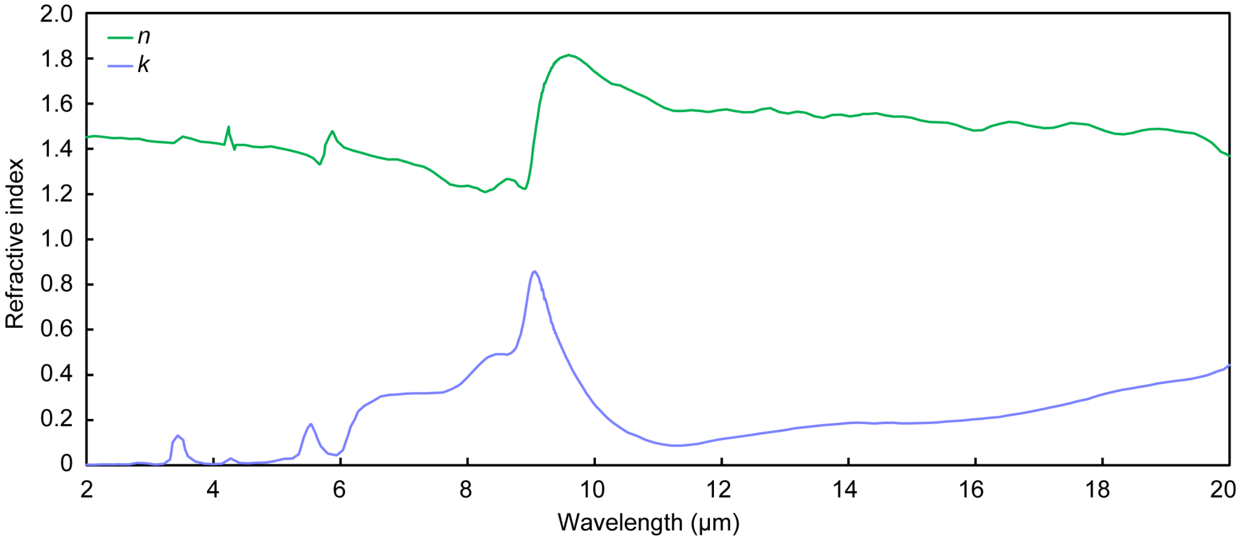

Figure S7. Experimentally measured complex refractive index spectra (*n* and *k*) of the SiO_2 NP–ETPTA composite (20 vol%) in the mid-infrared (MIR) range. These optical constants were utilized for the numerical calculations of mid-IR absorptivity/emissivity presented in Figure 2f and Figure 3d.

5. Monte Carlo simulation model for the analysis of photonic fiber.

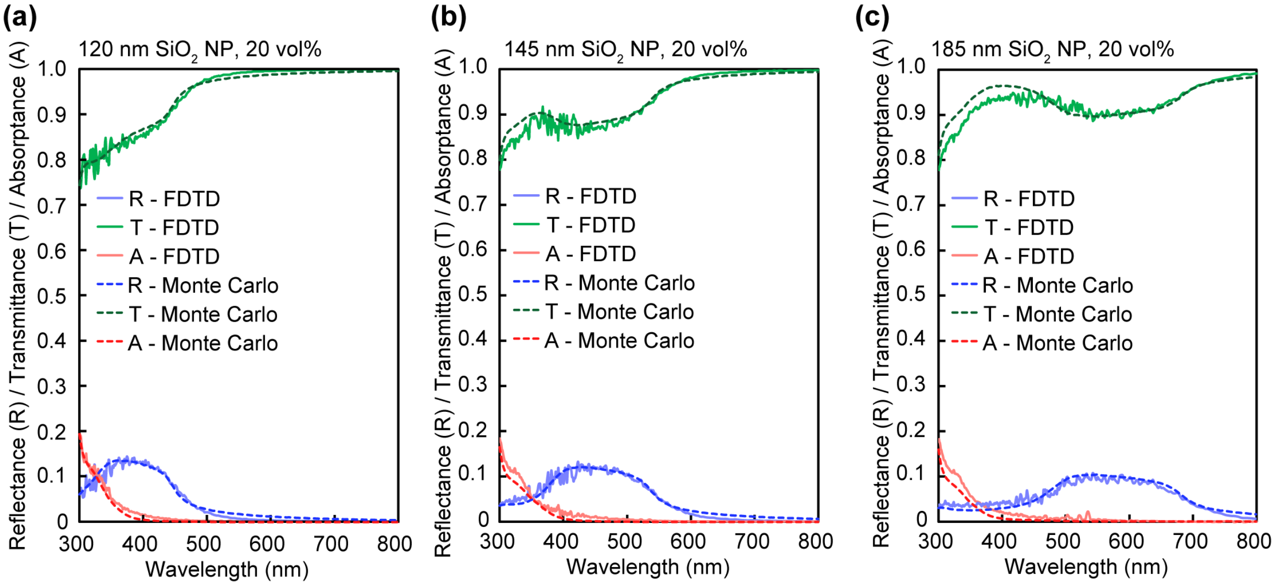

Figure S8. Validation of the Monte Carlo simulation model against FDTD calculations for planar photonic glass films with thickness of 32 μm. Comparison of the reflectance (R), transmittance (T), and absorptance (A) spectra for SiO_2-ETPTA composites with SiO_2 NP diameters of (a) 120 nm, (b) 145 nm, and (c) 185 nm. The solid lines represent full-wave FDTD results, while the dashed lines correspond to the Monte Carlo simulations. The Monte Carlo results are obtained through forward prediction using physical inputs identical to those established for the full wave FDTD simulations, including the complex refractive index, the NP diameters, and the filling fractions. A single broadband fine surface roughness parameter (0.5) is applied across the entire simulated range without any wavelength-by-wavelength tuning. The coarse surface is ignored based on the planar surface configuration of FDTD simulation model. The NPs were modeled as being fully embedded within the ETPTA matrix without any interface between air and SiO_2-ETPTA composites. The close agreement between the two methods confirms the accuracy of the Monte Carlo model for predicting the optical properties of the disordered medium.

To investigate the optical transport properties of cylindrical photonic glass fibers, a three dimensional Monte Carlo simulation was developed based on the previously reported photon packet Monte Carlo approach.[11] The custom python code used for this Monte Carlo simulation is provided in GitHub (https://github.com/NEOlab-code/Monte-Carlo-simulation-for-photonic-glass-fiber.git). Before applying this model to the complex fiber geometry, its accuracy was rigorously validated against full wave FDTD simulations using a planar SiO_2-ETPTA composite film configuration.

The disordered photonic glass medium was first treated as a homogeneous medium using the Bruggeman effective medium approximation. The scattering and extinction properties of the NPs in this effective medium were then calculated using the Aden Kerker solution to Mie theory. To take account the effect of structural correlation, the scattering of nanoparticles was coupled with the structure factor $S(q)$, which was obtained from an analytical solution for the measurable structure factor of a multispecies polydisperse Percus-Yevick fluid.[12] This led to the determination of a correlated scattering cross section and a correlated phase function. From these, the correlated scattering mean free path l_s, defined as the length at which the free-flight probability decays to $1/e$, was computed to simulate photon packet propagation through the film.

The simulation is initiated by launching photon packets from a virtual source plane located above the film. The initial photon packet is assigned a weight of 1, and the initial interaction with the surface is determined by the Fresnel equations. A coarse roughness parameter that slightly tilts the local surface

normal was introduced to account for random surface roughness. A random number determines whether the photon is reflected or refracted into the film. If reflection occurs, the simulation for that packet terminates and its weight is added to the total reflectance.

If the photon is refracted, it enters the transport phase where the path length for each step is sampled from an exponential distribution based on the l_s. Here, a fine roughness parameter was introduced to account for NPs on the top surface of the film. If a random number exceeds the fine roughness parameter, the correlated l_s is used; otherwise, the l_s calculated from single NP scattering is employed.

During transport within the film, the correlated l_s is used for path length determination, and the scattering direction is updated at each step by sampling from the correlated phase function. As the photon packet travels, its weight is updated to reflect the intensity loss due to absorption within the effective medium. Following the Beer Lambert law, the intensity is multiplied by the exponential decay factor associated with the absorption coefficient and the traveled path length.

This iterative process of determining path length, updating direction, and decaying the weight continues until the photon packet meets a termination condition. The simulation ends if the packet escapes through the top surface (contributing to reflectance), escapes through the bottom surface (contributing to transmittance), or if its weight falls below a specific threshold (e.g., 10^{-10}), at which point it is considered fully absorbed. For packets that exit through reflection or transmission, the difference between the initial and final weight is added to the total absorptance to ensure energy conservation.

For all numerical results, we employed 500,000 photon packets per wavelength to ensure statistical convergence and high spectral resolution. For simulations of the SiO_2 NPs in ETPTA (20% volume fraction), the colloids were modeled as an ETPTA shell and a SiO_2 core. This approach allows the disordered colloidal packing to be generated using the Percus-Yevick approximation while maintaining the relatively low volume fraction of the SiO_2 NPs. The refractive indices of the materials presented in **Figure S3** were used throughout. Except the coarse and fine roughness, all parameters were determined from the analytical solutions for structural configuration, scattering, extinction, and light propagation without any fitting based on the full wave numerical simulations or experimental measurements. As illustrated in **Figure S8**, the calculated reflectance, transmittance, and absorptance spectra exhibit high consistency with the FDTD results across all particle sizes (120 nm, 145 nm, and 185 nm), verifying the precision of the Monte Carlo algorithm.

For the fiber array simulation, a periodic arrangement of infinite cylinders was modeled by employing periodic boundary conditions (PBC) along the lateral direction to replicate the geometry of a tightly packed fiber. For this simulation, we define imaginary planes at the top and bottom sides of the fiber array to count the reflected or transmitted photon packets with corresponding termination conditions. The general simulation logic and process are identical to the planar film calculation except for the PBC treatment and the modified termination conditions of the photon packets. For the angle resolved reflection spectra analysis, photons escaping through the upper boundary are analyzed for their exit trajectories. The exit direction vector is converted into spherical coordinates, and a photon is recorded as detected only if its trajectory falls within a specific angular aperture defined by the acceptance angle of the detector and its position relative to the light source.

6. Synthesis of monodisperse silica NPs

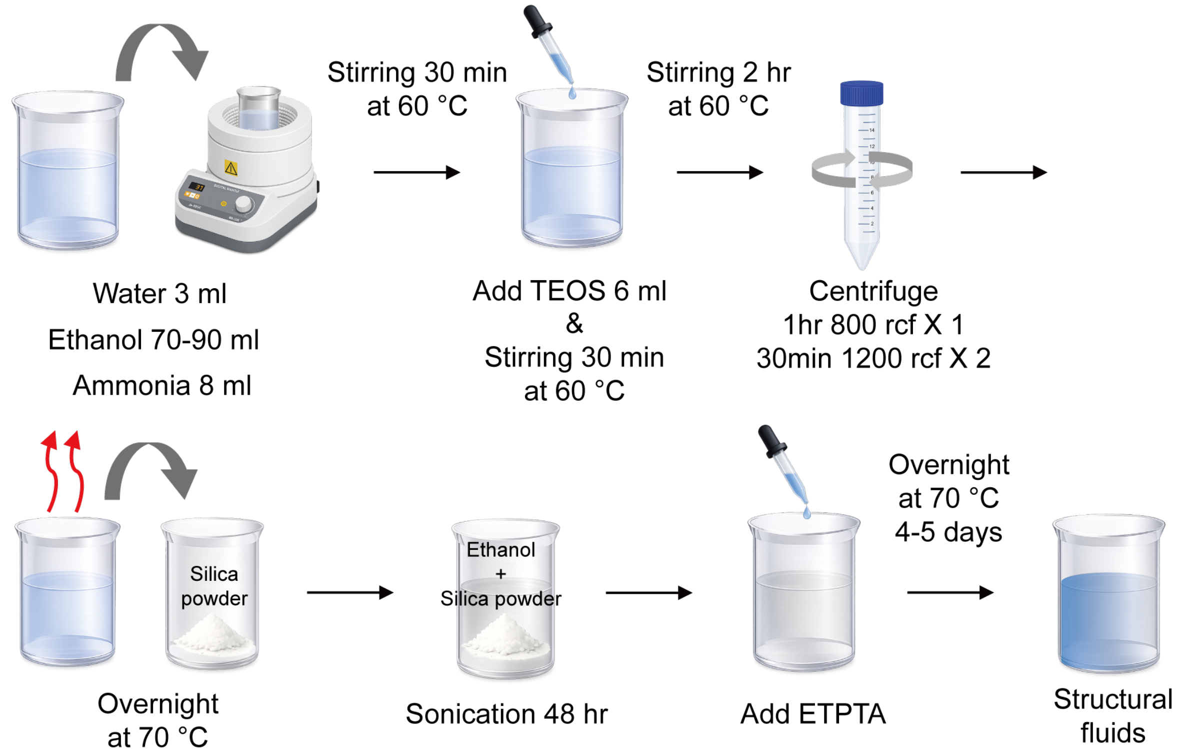

Figure S9. Schematic illustration of the high monodispersity of SiO_2 NP synthesis and preparation process of SiO_2 NP structural fluids.

A sol–gel reaction was employed to synthesize highly monodisperse SiO_2 NPs via a modified Stöber method. Specifically, 3 mL of deionized water, 70–90 mL of ethanol (the volume adjusted to control particle size), and 8 mL of 25 wt% ammonia solution were mixed and stirred at 60 °C for 30 min. Subsequently, 6 mL of tetraethyl orthosilicate (TEOS) was added as a silica precursor and the mixture was further stirred at 60 °C for 2 h to complete particle formation. The resulting colloidal suspension was centrifuged (800 × g for 1 h, followed by 1200 × g for 30 min × 2) and washed with ethanol. The collected particles were dried overnight in a 70 °C oven to obtain silica powder. The resulting silica powder was then weighed and its volume was determined using a density of 2.04 g/cm^3. For structural fluid preparation, 1.0–1.5 g of silica powder was redispersed in ethanol by ultrasonication for 48 h, followed by the addition of ETPTA resin in a controlled volumetric ratio. To account for the 4% volume shrinkage of ETPTA during polymerization,[13] the initial volume fraction of SiO_2 NPs in the ETPTA monomer was adjusted to approximately 19 vol% to achieve a final volume fraction of 20 vol% after UV curing. The resulting mixture was aged at 70 °C for 4–5 days to yield homogeneous SiO_2–ETPTA colloidal structural fluids suitable for microfluidic extrusion.

7. Microfluidic chip

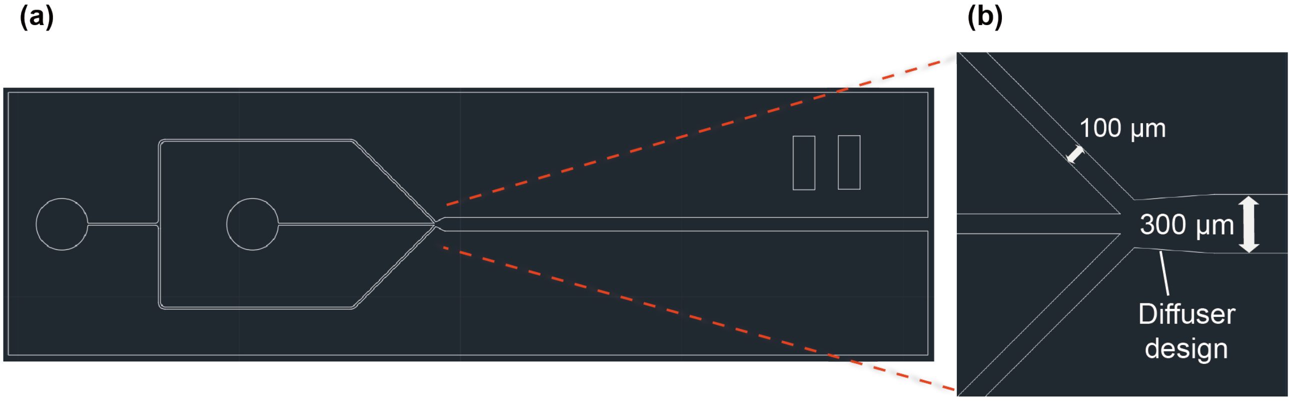

Figure S10. (a) CAD schematic of the designed microfluidic T-junction chip used for fiber fabrication. (b) Magnified view of the junction region, where two inlet channels (width = 100 µm) meet and merge into a single outlet channel (width = 300 µm). To maintain stable flow focusing and balanced shear forces during extrusion, a diffuser-type geometry was incorporated at the junction, enabling smooth pressure transition and consistent structural-fluid formation within the microchannel.

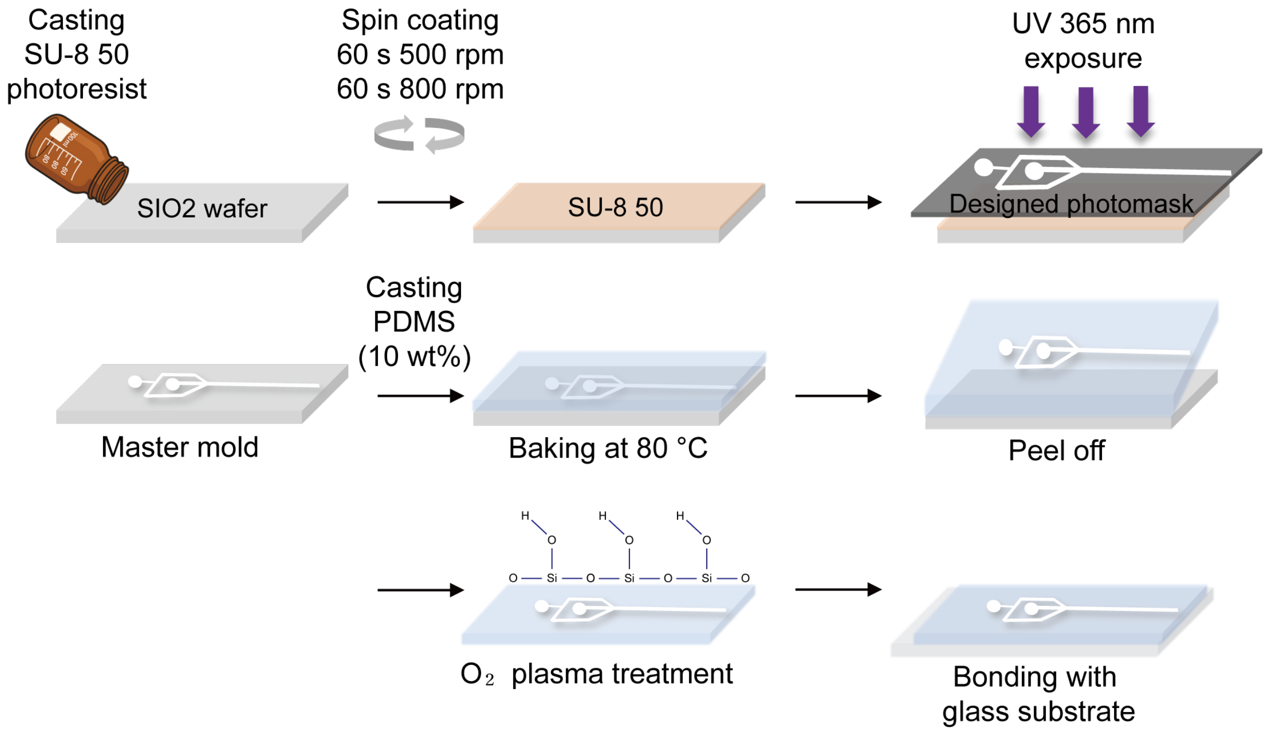

Figure S11. Schematic illustration of the soft lithography process used to fabricate the T-junction microfluidic chip.

A layer of SU-8 50 photoresist was spin-coated onto a SiO_2 wafer (500 rpm for 60 s, followed by 800 rpm for 60 s) and selectively exposed to 365 nm UV light through a CAD-designed photomask to define the microchannel pattern. After development, the resulting SU-8 master mold was used to cast PDMS (10 wt %) prepolymer mixed with curing agent, followed by thermal curing at 80 °C to obtain a PDMS channel mold. The cured PDMS replica was peeled off, and both the PDMS surface and a glass substrate were treated with O_2 plasma to activate surface bonding. Finally, the PDMS mold was

aligned and irreversibly bonded to the glass substrate, completing the T-junction microfluidic chip fabrication.

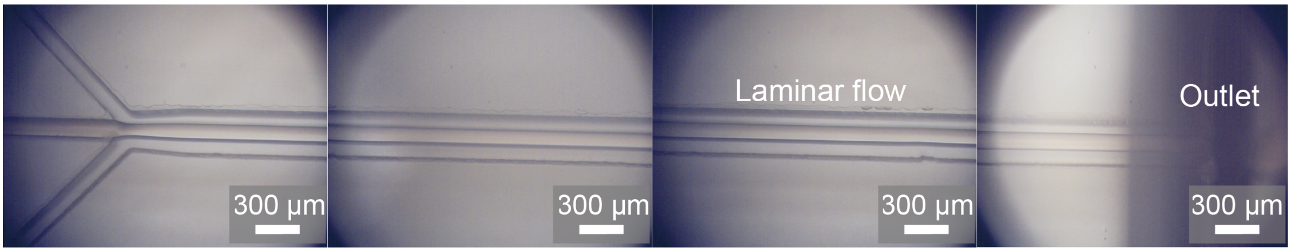

Figure S12. Optical microscope images of T-junction chip during the fiber extrusion operation. SiO_2 NP–ETPTA structural-fluid core stream and the 15 wt% PVA aqueous sheath flow are observed to merge and maintain a stable laminar flow throughout the channel length. This stable interface ensures uniform shear distribution and continuous formation of photonic glass fibers with consistent diameter and smooth morphology at the outlet.

8. Areal factor of assembled textile

We quantified fiber areal factor and porosity from RGB photographs using a compact, deterministic pipeline (Python; OpenCV, scikit-image, scikit-learn, numpy, and matplotlib). To suppress low-frequency shading, images were converted to CIELAB and the lightness channel L was flattened by subtracting a morphological opening with an elliptical structuring element of radius r. The result was linearly rescaled to [0,255][14,15]:

$$L_{flat} = rescale(L - (L \circ B_r) \qquad \text{(Equation S6)}$$

Where o denotes morphological opening and B_r is the disk/ellipse of radius r.

The fiber mask $F \subset I$ (with I the image domain) was produced by a fusion of a local Sauvola thresholding and a global Otsu threshold computed on L_{flat}. The Sauvola local threshold T_s with window size w and sensitivity k is

$$T_s(x,y) = m(x,y)\left[1 + k(\frac{s(x,y)}{R} - 1)\right] \qquad \text{(Equation S7)}$$

where $m(x,y)$ and $s(x,y)$ are the local mean and standard deviation within a $w \times w$ window and R is the dynamic range.[16] The Otsu threshold T_o maximizes the between-class variance $\sigma_B^2(T)$ of the histogram of L_{flat}[17]:

$$T_o = argmax\ \sigma_B^2(T) \qquad \text{(Equation S8)}$$

We fused the decisions with a logical OR to reduce false negatives:

$$F = \left(L_{flat} > T_s(w,k)\right) \vee \left(L_{flat} > T_O\right) \qquad \text{(Equation S9)}$$

From the final mask F we reported areal factor and porosity as

$$AF(\%) = 100 \times \frac{F}{I} \qquad \text{(Equation S10)}$$

$$Porosity(\%) = 100 - AF \qquad \text{(Equation S11)}$$

For optional uniformity assessment, the field was partitioned into N equal vertical bands and into a 2x 2 patch grid; per-region fiber fractions, and their mean.

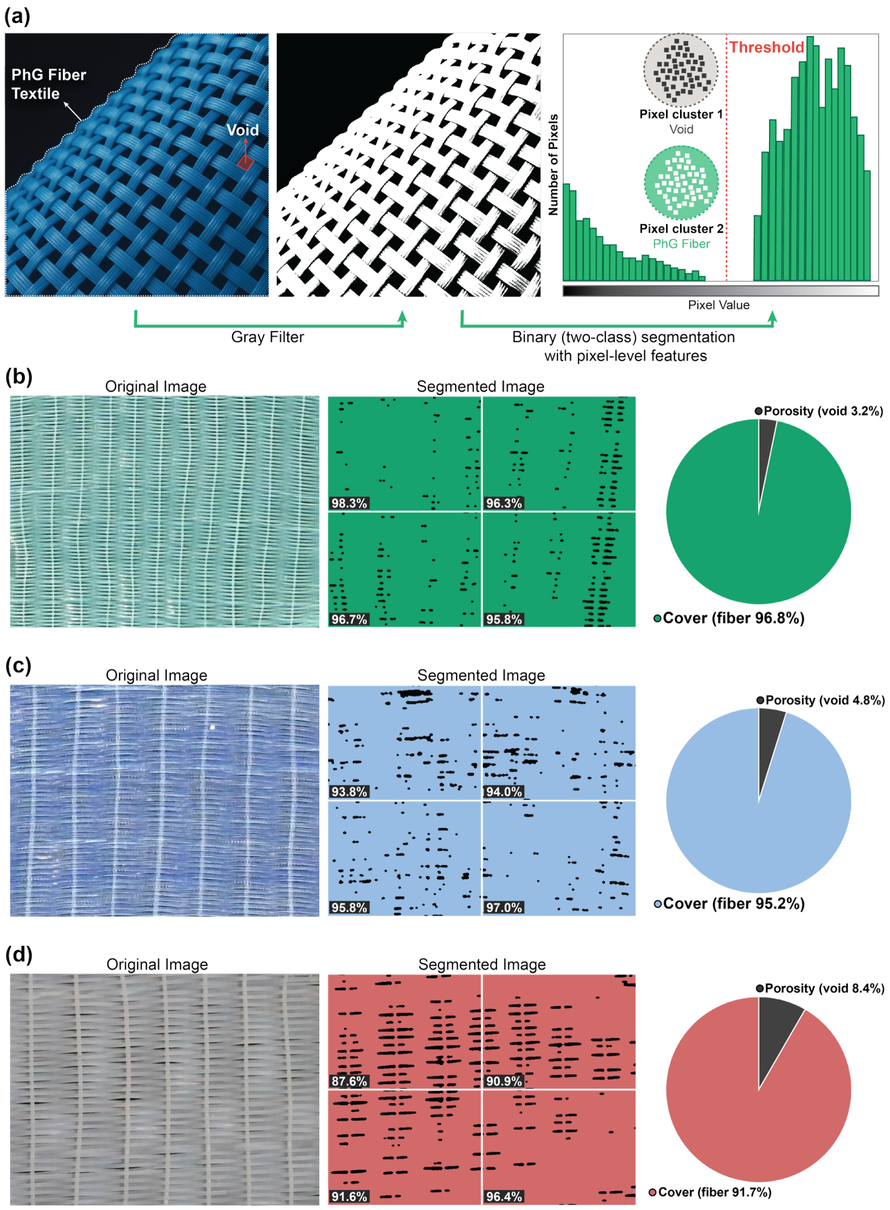

Figure S13. Quantitative image-segmentation of the photonic glass fiber textiles. (a) Image processing scheme demonstrating the conversion to grayscale and binary segmentation based on pixel intensity. An image of a woven PhG textile is converted to a fiber mask and the fiber areal factor is computed as the fraction of image pixels assigned to fiber; the complement is porosity (void). Representative results for (b) green, (c) blue, and (d) red PhG textiles. Original cropped image (left panel), fiber mask rendered in the sample over a 2 x 2 grid with the per-tile fiber fractions (middle panel), and a pie chart summarizing areal cover (fiber %) versus porosity (void %) (right panel).

9. Details of simulated skin

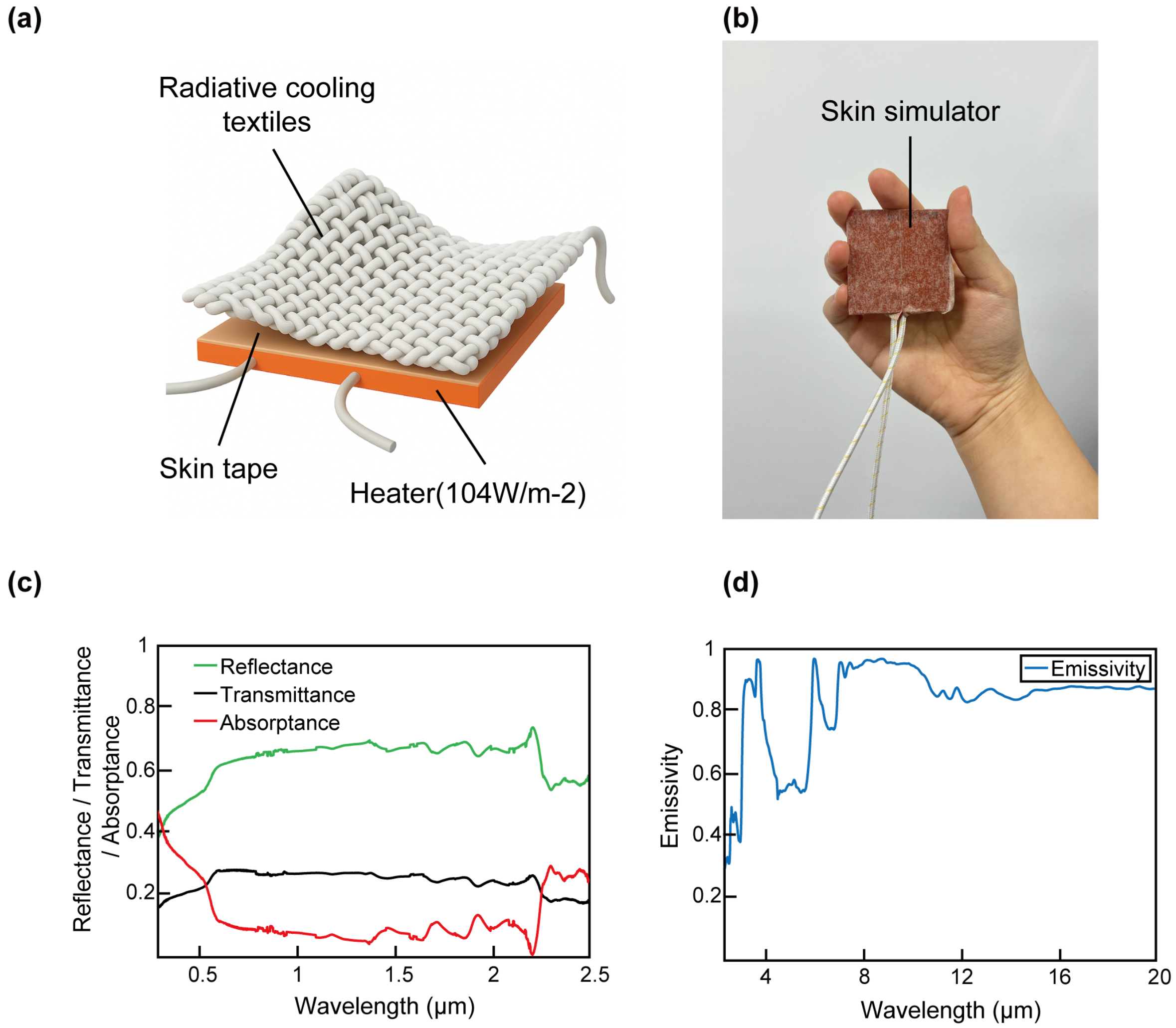

Figure S14. Details of the simulated skin. (a) Schematic of the skin simulator. (b) Photograph of the fabricated skin simulator. (c, d) Optical characterization of the skin simulator in the visible to near-IR (c) and MIR (d) ranges.

A skin-mimicking substrate was fabricated to reproduce both the optical and thermal characteristics of human skin (**Figure S14**). The outermost layer consisted of a 3M Micropore™ medical tape (1533-1), which exhibits visible–near-infrared (Vis–NIR) reflectance and transmittance comparable to those of real human skin (**Figure S14c**). In the mid-infrared (mid-IR) range, the artificial skin shows high and broadband emissivity across the atmospheric transparency window (**Figure S14d**), making it an appropriate surrogate for epidermal thermal radiation.

A silicone rubber heater was attached beneath the skin tape and operated at a power density of 104 W m^{-2}, corresponding to the average basal metabolic heat flux of the human body. The heater temperature was maintained at approximately 36 °C (310 K), establishing a steady thermal baseline representative of human skin under resting conditions.

10. Indoor radiative cooling performance

To quantitatively interpret the temperature reduction observed for the photonic-glass textiles in the indoor solar-simulator experiment (**Figure 7**), an approximate cooling-power analysis was performed based on a simplified energy-balance framework. This analysis converts the measured surface temperature differences between bare skin and textile-covered skin into an effective cooling power per unit area.

Skin-simulator properties and experimental assumptions

Detailed information for optical properties at solar spectrum and mid-IR properties are provided in previous section (**Figure S14c-S14d**). During the indoor solar-simulator measurements, no fan or forced airflow was applied, and therefore forced convection can be neglected. The thermal exchange at the skin surface is assumed to arise from (i) natural convection and (ii) thermal radiation with the surrounding indoor environment. Because the experiment was conducted indoors, the samples did not have a view factor to the cold sky; instead, they predominantly exchanged radiation with surrounding laboratory walls and instrument housings at near-ambient temperatures.

Energy-balance model

Under quasi-steady conditions, the surface energy balance of the skin simulator can be expressed as

$$q_{in} - q_{out} = 0 \qquad \text{(Equation S12)}$$

where q_{in} and q_{out} denote the net incoming and outgoing heat fluxes, respectively. For two samples measured under identical environmental conditions (bare skin vs. textile-covered skin), the difference in net heat flux can be approximated by a linearized heat-transfer relation:

$$\Delta P_{cool}(t) \approx h_{eff} \cdot [T_{bare}(t) - T_{textile}(t)] \qquad \text{(Equation S13)}$$

where ΔP_{cool} is the additional cooling power per unit area provided by the textile, T_{bare} and $T_{textile}$ are the measured surface temperatures of the bare skin and textile-covered skin, respectively, and h_{eff} is an effective heat-transfer coefficient.

The effective coefficient h_{eff} includes contributions from natural convection (h_{conv}) and linearized thermal radiation ($h_{rad,lin}$):

$$h_{eff} = h_{conv} + h_{rad,lin} \qquad \text{(Equation S14)}$$

We report the convective heat transfer coefficient and the corresponding convective heat dissipation for both the draft-shielded (LDPE film) measurement condition and the open-air measurement condition separately. In particular, for indoor measurements (performed without forced airflow), the convective heat transfer coefficient is typically modeled by using a standard range of h_{conv} = 5–10 W m^{-2} K^{-1}.[15,16] For outdoor measurements, the convective coefficient can vary depending on wind and local airflow; therefore, we report cooling powers using a representative range as described in Section 11.

$$h_{rad,lin} \approx 4\varepsilon\sigma T_m^3 \qquad \text{(Equation S15)}$$

where ε is the effective mid-IR emissivity of the surface, σ is the Stefan–Boltzmann constant, and T_m is the mean surface temperature. Using $\varepsilon \approx 0.9$ (consistent with the measured mid-IR emissivity

of the artificial skin and textiles) and $T_m \approx 310\ K$ yields $h_{rad,lin} \approx 5-6\ W\ m^{-2}\ K^{-1}$. Accordingly, a representative range of $h_{eff} \approx 10-15\ W\ m^{-2}\ K^{-1}$ is adopted for the present analysis.

Heat-transfer model in textiles

The corresponding cooling-power ranges for both indoor and outdoor experimental conditions were then computed using Eq. S16–S21.

$$\Delta P_{\text{cool}}(t) = P_{\text{gen}}^{(\text{bare})}(t) + P_{\text{solar}}^{(\text{bare})}(t) + P_{\text{ir,gain}}^{(\text{bare})}(t) - P_{\text{ir,loss}}^{(\text{bare})}(t) - P_{\text{non-rad}}^{(\text{bare})}(t) \quad \text{(Equation S16)}$$

In this expression, $P_{\text{gen}}^{(\text{bare})}(t)$, $P_{\text{solar}}^{(\text{bare})}(t)$, $P_{\text{ir,gain}}^{(\text{bare})}(t)$, $P_{\text{ir,loss}}^{(\text{bare})}(t)$, and $P_{\text{non-rad}}^{(\text{bare})}(t)$ correspond to the internally generated heat flux, absorbed solar power, infrared radiative gain from the environment, infrared radiative loss from the skin surface, and non-radiative heat transfer, respectively.

The internally generated heat flux supplied by the skin phantom was fixed as

$$P_{\text{gen}}^{(\text{bare})}(t) = 104 \text{W m}^{-2} \quad \text{(Equation S17)}$$

corresponding to the electrical heating power normalized by the exposed area.

The absorbed solar heat flux at the skin surface beneath the textile is given by

$$P_{\text{solar}}^{(\text{bare})}(t) = q_{\text{solar}}\, \tau_{\text{solar,textile}}\left(1-\rho_{\text{solar,bare}}\right) \sum_{n=0}^{\infty} \left(\rho_{\text{solar,bare}}\, \rho_{\text{solar,textile}}\right)^{n} \quad \text{(Equation S18)}$$

where q_{solar} is the incident solar irradiance, $\tau_{\text{solar,textile}}$ is the solar transmittance of the textile, and $\rho_{\text{solar,bare}}$ and $\rho_{\text{solar,textile}}$ are the solar reflectances of the bare skin phantom and the textile, respectively. The summation term accounts for multiple internal reflections between the skin surface and the textile.

The infrared radiative heat gained by the skin surface from the environment is expressed as

$$P_{\text{ir,gain}}^{(\text{bare})}(t) = \varepsilon_{\text{bb,textile}}\, \varepsilon_{\text{bb,bare}}\, \tau_{\text{textile,ir}}\, T_{\text{amb}}^{4} + \varepsilon_{\text{bb,textile}}\, \varepsilon_{\text{bb,bare}}\left(1-\tau_{\text{bb,atm}}\right) T_{\text{amb}}^{4} \quad \text{(Equation S19)}$$

where $\varepsilon_{\text{bb,bare}}$ and $\varepsilon_{\text{bb,textile}}$ are the effective broadband infrared emissivities of the bare skin phantom and the textile, respectively, $\tau_{\text{textile,ir}}$ is the infrared transmittance of the textile, $\tau_{\text{bb,atm}}$ is the atmospheric infrared transmittance, and T_{amb} is the ambient temperature.

The infrared radiative heat loss from the skin surface is given by

$$P_{\text{ir,loss}}^{(\text{bare})}(t) = \left(1-\rho_{\text{textile,bb}}\right)\sigma \varepsilon_{\text{bb,bare}} T_{\text{bare}}^{4} \quad \text{(Equation S20)}$$

where $\rho_{\text{textile,bb}}$ is the broadband infrared reflectance of the textile, σ is the Stefan–Boltzmann constant, and T_{bare} is the surface temperature of the bare skin phantom.

The non-radiative heat transfer due to convection and conduction across the air gap between the skin phantom and the textile is modeled as

$$P_{\text{non-rad}}^{(\text{bare})}(t) = h_{eff} \cdot [T_{bare}(t) - T_{textile}(t)] \qquad \text{(Equation S21)}$$

where h_{eff} is the effective non-radiative heat-transfer coefficient, incorporating natural convection and linearized radiative contributions, and T_{textile} denotes the inner-surface temperature of the textile adjacent to the skin phantom.

Cooling power in the dark state (before illumination)

Even before the solar simulator was turned on, the textile-covered skin samples exhibited lower surface temperatures than the bare-skin reference (**Figure 7b**). Immediately prior to illumination (t ≈ 6 min), the approximate temperatures were:

$T_{bare} \approx 38.25\ °C$
$T_{blue\ textile} \approx 37.25\ °C\ (\Delta T \approx 1.00\ K)$
$T_{green\ textile} \approx 36.08\ °C\ (\Delta T \approx 2.17\ K)$
$T_{red\ textile} \approx 37.41\ °C\ (\Delta T \approx 0.84\ K)$

Using the effective heat-transfer coefficient range above, the corresponding dark-state cooling powers are estimated as:

Blue textile: $\Delta P_{cool} \approx 10 - 15\ W\ m^{-2}$
Green textile: $\Delta P_{cool} \approx 22 - 33\ W\ m^{-2}$
Red textile: $\Delta P_{cool} \approx 8 - 13\ W\ m^{-2}$

Although the absolute temperatures of all samples gradually increase with time in the dark, this behavior is attributed to net radiative heat exchange with the indoor surroundings rather than solar absorption. In the absence of a cold-sky radiative sink, high-emissivity surfaces exchange thermal radiation with nearby warm objects, leading to a gradual temperature rise even under dark conditions. Nevertheless, the textile-covered samples consistently exhibit a lower net heat gain than bare skin.

Cooling power under solar illumination

After the solar simulator was turned on, all samples experienced additional heating due to solar irradiation. However, the textile-covered skins remained consistently cooler than the bare-skin reference throughout the measurement. At $t \approx 60$ min, the measured temperatures were approximately:

$T_{bare} \approx 56.14\ °C$
$T_{blue\ textile} \approx 52.02\ °C\ (\Delta T \approx 4.12\ K)$
$T_{green\ textile} \approx 51.99\ °C\ (\Delta T \approx 4.15\ K)$
$T_{red\ textile} \approx 51.27\ °C\ (\Delta T \approx 4.86\ K)$

These temperature differences correspond to estimated cooling powers of:

Blue textile: $\Delta P_{cool} \approx 41 - 62\ W\ m^{-2}$
Green textile: $\Delta P_{cool} \approx 42 - 62\ W\ m^{-2}$
Red textile: $\Delta P_{cool} \approx 49 - 73\ W\ m^{-2}$

Under solar illumination, this enhanced cooling is primarily attributed to reduced solar heat absorption by the skin due to strong scattering and reflection of visible and near-infrared radiation by the photonic-glass textiles, while maintaining high and broadband mid-infrared emissivity for thermal radiation.

11. Details of outdoor cooling power calculations

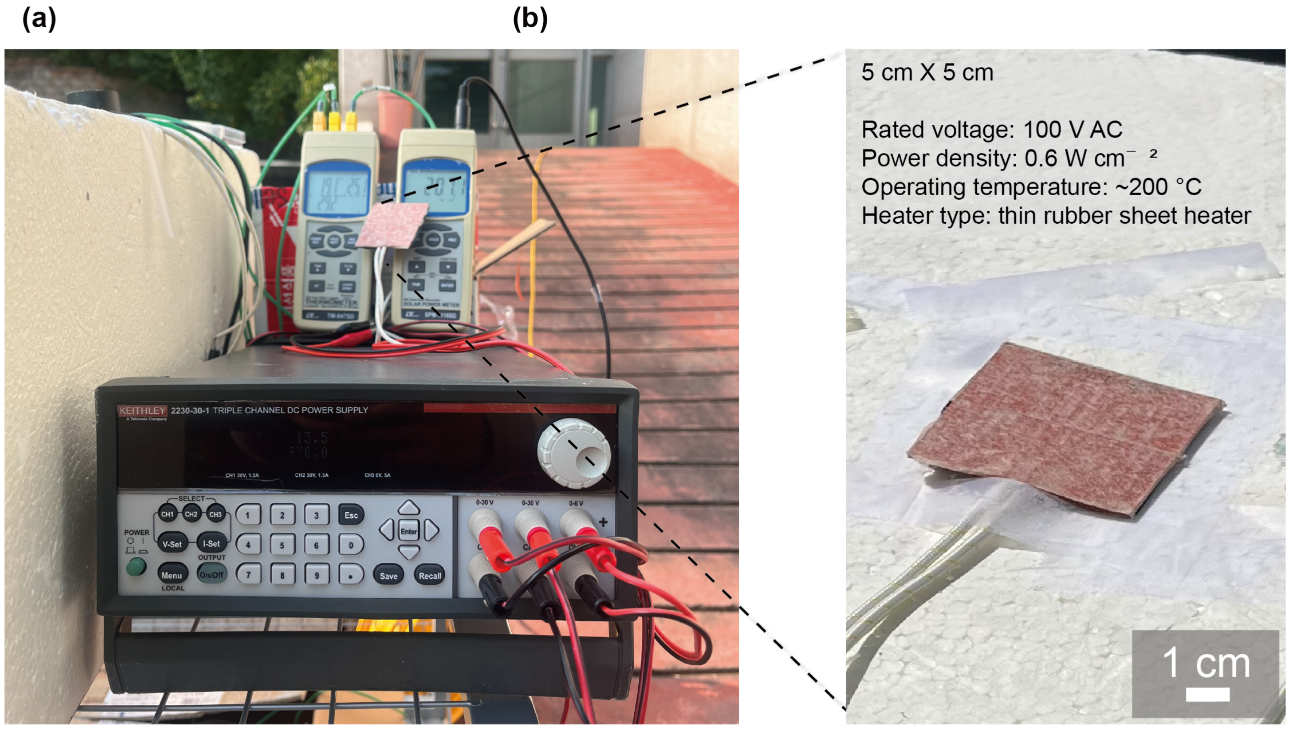

Figure S15. Calculation of Required Voltage for the Simulated Bare Skin Test. (a) Photograph of the experimental setup used for the simulated bare-skin heating test. A 5 cm × 5 cm thin-film heater was embedded beneath a skin-mimicking silicone phantom and operated using a programmable DC power supply connected to an external data logger. (b) Calculation workflow used to determine the required heater voltage to reproduce the average human-body radiative heat flux. (c) The operating voltage (≈13 V) was selected to generate a surface heat flux of ≈104 W m^{-2}, corresponding to the natural radiative emission of human skin at ~36 °C.

This note describes (i) the calibration of the skin-mimicking phantom to reproduce the baseline radiative heat flux of human skin and (ii) the procedure used to extract cooling-power values from the 24 h outdoor measurements shown in **Figure 8**.

Calibration of the skin-mimicking heat flux (Figure S15): A 5 cm × 5 cm skin phantom (area A = 0.0025 m^2) was heated using a thin rubber sheet heater embedded beneath a skin-mimicking surface layer. The target basal heat flux was set to $q = 104\ W\ m^{-2}$, which corresponds to the average radiative heat generation of resting human skin near 36 $°C$ (310 K). The required total heating power is therefore[18]

$$P_{req} = q \times A = (104\ W\ m^{-2}) \times (0.0025\ m^2) = 0.26 \qquad \text{(Equation S22)}$$

The commercial heater is rated at $V_{rated} = 100\ V$ and $P_{rated} = 15\ W$. Assuming ohmic behavior (constant electrical resistance), the delivered electrical power scales with the square of the applied voltage. Thus, the operating voltage required to deliver P_{req} is[18]

$$V_{req} = V_{rated} \times (P_{req} / P_{rated})^{0.5} \qquad \text{(Equation S23)}$$

Substituting the parameters gives $V_{req} \approx 13.1\,V$. Accordingly, an input voltage of $\sim 13\,V$ was applied during the simulated bare-skin tests to reproduce a surface heat flux of $\sim 104\ W\ m^{-2}$.

Quality check: $V_{rated} = 100\,V$ and $P_{rated} = 15\,W$ imply an effective heater resistance of $R \approx {V_{rated}}^2/P_{rated} = 10000/15 \approx 667\,\Omega$. At $V_{req} \approx 13.1\,V$, the expected power is $P = {V_{req}}^2/R \approx 0.26\,W$, consistent with Eq. S16–S17. Note that any mismatch between AC/DC ratings is negligible for a purely resistive heater; the calibration is based on electrical power delivery (V^2/R).

Cooling-power definition for outdoor tests (Figure 8): Outdoor tests were performed with each 5 cm × 5 cm textile mounted on the heated skin phantom. Surface temperature was recorded continuously together with solar irradiance and humidity (**Figure 8c–d**). The "ΔP_{cool}" reported in **Figure 8e** is defined as the net reduction in heat load at the skin surface provided by the textile relative to the bare-skin reference under the same environmental conditions.

For reference, the meteorological ambient air temperature outside the enclosure on 10 September 2025 in Seoul ranged from 27–32 °C during 11:00–15:00 and from 19–22 °C during 23:00–03:00, based on the Seoul/Kimpo International Airport weather station. The air-temperature sensor in our setup was located inside the wind-shielded enclosure to capture the local convective environment around the samples; this local 'enclosure air temperature' can exceed the meteorological ambient temperature under direct solar illumination due to greenhouse heating.

To translate the measured temperature reduction into a cooling power per unit area, we adopt a lumped energy-balance model in which the skin surface exchanges heat with the environment via natural convection and thermal radiation. For two samples measured side-by-side (bare vs. textile-covered), as aforementioned the additional cooling power is approximated as $\Delta P_{cool}(t) \approx h_{eff} \cdot [T_{bare}(t) - T_{textile}(t)]$, where $h_{eff} = h_{conv} + h_{rad,lin}$, with $h_{rad,lin} \approx 4\varepsilon\sigma {T_m}^3$.

Here, ε is ≈ 0.9, consistent with the measured emissivity of the photonic-glass textiles and the skin-mimicking layer. For $T_m \approx 300 - 320\,K$, $h_{rad,lin}$ is $\sim 5 - 6\ W\ m^{-2}\ K^{-1}$. No fan or forced airflow was applied during the outdoor test; therefore, h_{conv} is governed by natural convection and wind-dependent fluctuations. In the absence of direct wind-speed measurement, we report cooling powers using a representative range $h_{eff} = 10 - 15\ W\ m^{-2}\ K^{-1}$ to provide an order-of-magnitude estimate and facilitate comparison across samples.

Table S1. Cooling-power ranges (ΔP_{cool}) extracted from the outdoor tests (**Figure 8e**) for the B, G, and R photonic-glass textiles, relative to the bare-skin phantom baseline, during daytime (11:00–15:00) and nighttime (23:00–03:00) windows.

Time window	Blue (B)	Green (G)	Reddish-white (R)
Daytime (11:00–15:00)	16–25 W m^{-2}	28–42 W m^{-2}	44–65 W m^{-2}
Nighttime (23:00–03:00)	28–42 W m^{-2}	28–42 W m^{-2}	30–46 W m^{-2}

In daytime (11:00–15:00), the cooling hierarchy follows R > G > B, consistent with the solar-band scattering/reflectance trends of the textiles. At night (23:00–03:00), all textiles maintain cooling relative to the bare-skin phantom; small differences among colors can arise from variations in textile openness/thickness (affecting the effective non-radiative heat transfer coefficient) and slight emissivity variations. Importantly, the outdoor trends reported in Figure 8 and Table S1 are based on side-by-side differential comparisons (bare vs. textile-covered) measured under identical conditions, which mitigates systematic temperature offsets.

To assess the magnitude of possible nighttime temperature variations, it should be noted that in the absence of solar input, the steady-state temperature rise of the heated phantom scales approximately as $\Delta T \approx q_{in}/(h_{conv} + h_{rad})$. Therefore, a modest variation in h_{conv} by $\sim 2 - 3\ W\ m^{-2}\ K^{-1}$ (within natural-convection variability for porous textiles and setup-dependent airflow) can lead to a few-degree change in ΔT at $q_{\text{in}} \approx 104$ W m^{-2}, consistent with the minor color-dependent differences observed in the outdoor nighttime traces (Figure 8d).

References

[1] G. T. Vladisavljević, N. Khalid, M. A. Neves, T. Kuroiwa, M. Nakajima, K. Uemura, S. Ichikawa, I. Kobayashi, *Adv. Drug Deliv. Rev.* **2013**, 65, 1626–1663.

[2] D. Tabor, R. H. S. Winterton, Proc. R. Soc. Lond. A **1969**, 312, 435–450.

[3] V. Baranau, U. Tallarek, J. *Chem. Phys*. **2017**, 147, 224503.

[4] A. F. Oskooi, D. Roundy, M. Ibanescu, P. Bermel, J. D. Joannopoulos, S. G. Johnson, *Comput. Phys. Commun.* **2010**, 181, 687–702.

[5] G. Shang, L. Maiwald, H. Renner, D. Jalas, M. Dosta, S. Heinrich, A. Petrov, M. Eich, *Sci. Rep.* **2018**, 8, 7804.

[6] S. Lang, L. Maiwald, D. Jalas, H. Renner, A. Petrov, M. Eich, *arXiv*, **2018**, 1802.07058.

[7] N. W. Ashcroft, J. Lekner, Phys. Rev. 1966, 145, 83–90.

[8] M. H. W. Chan, K. I. Blum, S. Q. Murphy, G. K. S. Wong, *Phys. Rev. Lett.* **1963**, 10, 321.

[9] W. Ashcroft, J. Lekner, *Phys. Rev.* **1966**, 145, 83–90.

[10] P. W. Atkins, J. de Paula, *Atkins' Physical Chemistry, Springer, Berlin Heidelberg, 10th ed.* **2014**.

[11] V. Hwang, A. B. Stephenson, S. Barkley, S. Brandt, M. Xiao, J. Aizenberg, V. N. Manoharan, *Proc. Natl. Acad. Sci. U.S.A.* **2021**, *118*, e2015551118.

[12] M. Ginoza, M. Yasutomi, *J. Phys. Soc. Jpn*, **1999**, *68*, 2292-2297

[13] P. Jiang, J. F. Bertone, K. S. Hwang, V. L. Colvin, *Chem. Mater.* **1999**, *11*, 2132.

[14] X. Bai, F. Zhou, Y. Xie, *J. Electron. Imaging* **2008**, *17*, 030501.

[15] S. Singh, M. -A. Bary, T. R. Jones, A. E. Carpenter, *J. Microsc.* **2014**, *256*, 231.

[16] J. Sauvola, M. Pietikäinen, *Pattern Recognit.* **2000**, *33*, 225.

[17] N. Otsu, *IEEE Trans. Syst., Man, Cybern.* **1979**, *9*, 62.

[18] F. P. Incropera, D. P. DeWitt, T. L. Bergman, A. S. Lavine, *Fundamentals of Heat and Mass Transfer*, Wiley.
